# An Artificial Conversion of Roots into Organs with Shoot Stem Characteristics by Inducing Two Transcription Factors

**DOI:** 10.1016/j.isci.2020.101332

**Published:** 2020-07-14

**Authors:** Shigeru Hanano, Hajime Tomatsu, Ai Ohnishi, Koichi Kobayashi, Yuki Kondo, Shigeyuki Betsuyaku, Eiji Takita, Yoshiyuki Ogata, Keishi Ozawa, Kunihiro Suda, Tsutomu Hosouchi, Takahiro Nagase, Hideyuki Suzuki, Nozomu Sakurai, Hiroshi Masumoto, Hiroo Fukuda, Daisuke Shibata

**Affiliations:** 1 Kazusa DNA Research Institute, 2-6-7 Kazusa-Kamatari, Kisarazu, Chiba 292-0818, Japan; 2 Graduate School of Arts and Sciences, The University of Tokyo, 3-8-1 Komaba, Meguro-ku, Tokyo 153-8902, Japan; 3 Department of Biological Sciences, Graduate School of Science, The University of Tokyo, 7-3-1 Hongo, Bunkyo-ku, Tokyo 113-0033, Japan; 4 Japan Science and Technology Agency (JST), PRESTO, 4-1-8 Honcho, Kawaguchi, Saitama 332-0012, Japan; 5 The Kisarazu Laboratory, Graduate School of Life Sciences, Tohoku University, 2-6-7 Kazusa-Kamatari, Kisarazu, Chiba 292-0818, Japan

**Keywords:** Plant Biology, Plant Genetics, Plant Development

## Abstract

Somatic plant cells can regenerate shoots and/or roots or adventitious embryonic calluses, which may induce organ formation under certain conditions. Such regenerations occur via dedifferentiation of somatic cells, induction of organs, and their subsequent outgrowth. Despite recent advances in understanding of plant regeneration, many details of shoot induction remain unclear. Here, we artificially induced shoot stem-like green organs (SSOs) in *Arabidopsis thaliana* roots via simultaneous induction of two transcription factors (TFs), ARABIDOPSIS THALIANA HOMEOBOX PROTEIN 25 (ATHB25, At5g65410) and the B3 family transcription factor REPRODUCTIVE MERISTEM 7 (REM7, At3g18960). The SSOs exhibited negative gravitropism and differentiated vascular bundle phenotypes. The ATHB25/REM7 induced the expression of genes controlling shoot stem characteristics by ectopic expression in roots. Intriguingly, the restoration of root growth was seen in the consecutive and adjacent parts of the SSOs under gene induction conditions. Our findings thus provide insights into the development and regeneration of plant shoot stems.

## Highlights

• Co-induction of ATHB25 and REM7 produces shoot stem-like organs (SSOs) in roots
• SSOs exhibit negative gravitropism and differentiated vascular bundles
• Shoot- and root-specific genes are up- and down-regulated, respectively, in SSOs
• The restoration of normal root growth follows the SSO formation

## Graphical Abstract

**Figure:**
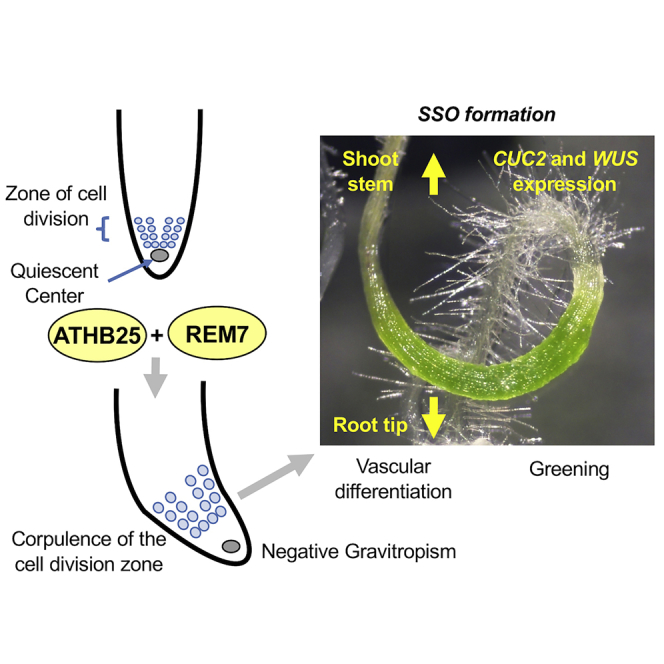

## Introduction

*De novo* organogenesis, so-called regeneration, is widely conserved in both animals and plants and functions to restore structures or organs damaged or lost by various physical assaults, such as injury, diseases, or attack by predators (Ikeuchi et al., 2016; Pulianmackal et al., 2014). Regenerative capabilities are particularly pronounced in plants, which can repair not only tissues and organs but also regenerate entirely new individual plants from damaged organs. The regeneration of organs is an essential step in biotechnological breeding and plant transformation protocols (Motte et al., 2014). In the initial process of regeneration, a pluripotent cell mass, termed a callus, is dedifferentiated from somatic cells, and the callus then induces formation of shoots and other organs upon treatment with certain phytohormones (Pulianmackal et al., 2014; Skoog and Miller, 1957). Recent studies have reported that callus formation resembles lateral root development processes, suggesting that root stem cell regulators induce callus regenerative to shoot initials (Ikeuchi et al., 2016; Sugimoto et al., 2010). However, details of the genetic background of shoot stem induction following callus formation remain largely unknown. Findings resulting from aberrant phenotypic phenomena generated by genetic manipulation could provide breakthroughs in understanding the genetic background of shoot stem formation, as reported regarding the molecular genetics of development of other organs. Here, we report the shoot stem induction activated by two transcription factors (TFs) expressed around the shoot apical meristems (SAMs) under normal growth conditions and subsequent restoration of root growth in the consecutive and adjacent parts of the shoot stem-like organs (SSOs) even under the conditions of the gene activation.

## Results

### Simultaneous Induction of ATHB25 and REM7 Generates Shoot Stem-like Green Organs

We selected 21 genes putatively encoding Arabidopsis TFs as candidate inducers of shoot stem formation, based on our hypothesis that such factors display SAM-specific expression (see Supplemental Information and Figures S1A–S1C, Table S1) (Doerner, 2003). We obtained nine full-length cDNAs from these candidates from the RIKEN BioResource Center (www.brc.riken.jp) (Seki et al., 2004) (Table S1, Figures S1A–S1C). These nine cDNAs were inserted between a chemically inducible promoter *Lex*A and terminator *hsp18.2* connected tandemly in the pDONR-based vector (Accession Number: LC217877) using the PRESSO method (Takita et al., 2013) and then transferred into the binary vector pGW501 (see Supplemental Information, Table S2). The gene construct was introduced into Arabidopsis plants, and expression of the genes was simultaneously induced in the roots of young seedlings using a ß-estradiol-mediated induction system (Zuo et al., 2000).

**Figure 1:**
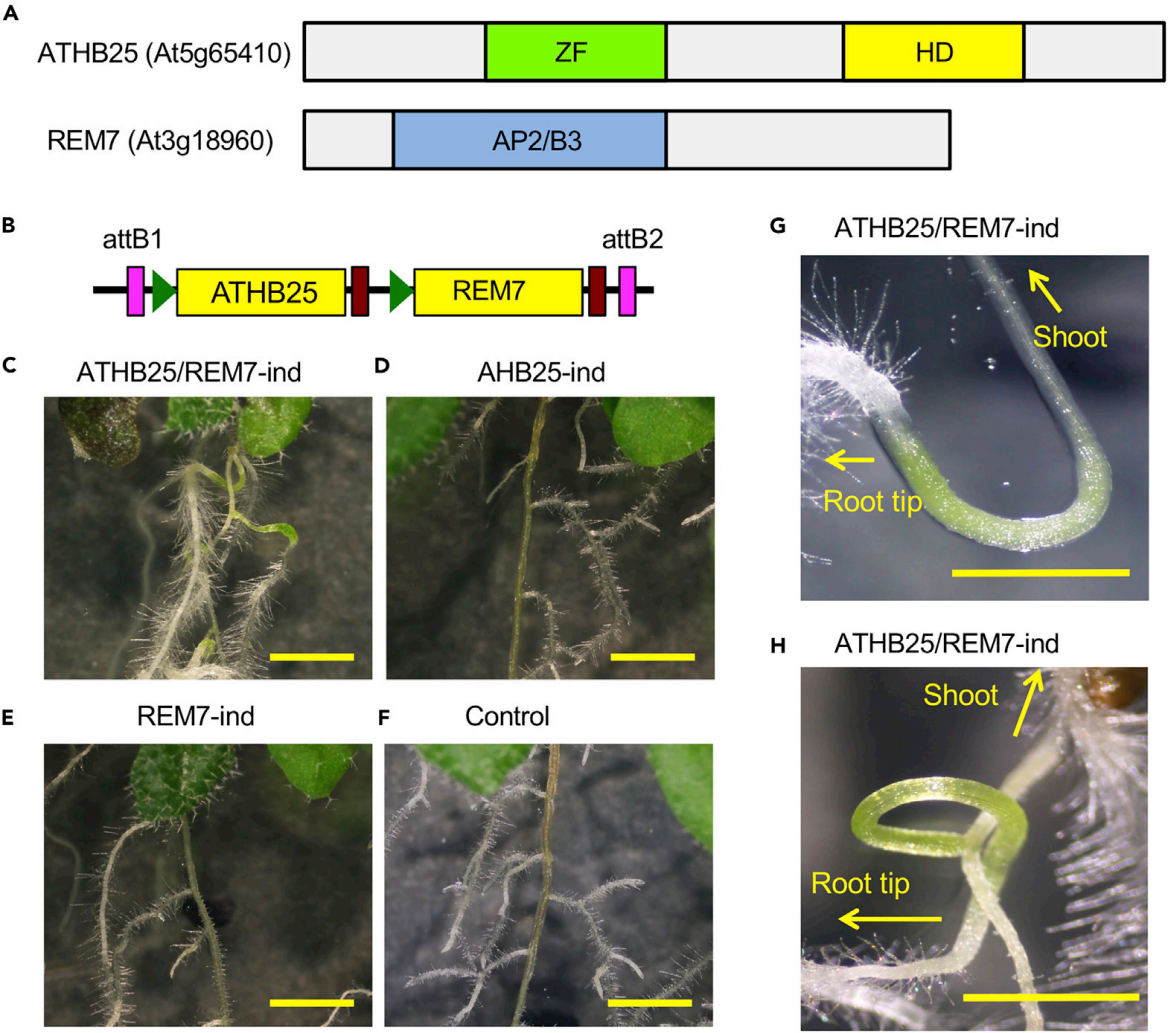
Simultaneous Induction of ATHB25 and REM7 Generates Shoot Stem-like Green Organs (SSOs) (A) Gene structures of *ATHB25* (*At5g65410*) and *REM7* (*At3g18960*). ZF, C2H2-type zinc finger domain; HD, homeodomain; and AP2/B3, AP2/B3-like DNA binding domain. (B) A construct for simultaneous induction of ATHB25 and REM7 (ATHB25/REM7-ind). Green triangles, yellow boxes, and brown boxes represent the XVE operator, coding regions, and terminator, respectively. Pink boxes indicate attB1 and attB2 sequences for Gateway cloning. (C–F) The roots of the ATHB25/REM7-ind (C), ATHB25-ind (D), REM7-ind (E), and the control (F) plants in 12 days after the induction. (G and H) SSOs formed in the main (G) and lateral (H) roots of the ATHB25/REM7-ind plant in 12 days after the induction. Scale bars are 2 mm (C–F) and 1 mm (G and H). See also Figures S1–S5.

Plants harboring the nine-TF-cDNA construct exhibited upward-elongated root caps following exposure to inductive conditions, and then the direction of root extension returned downward (Figures S1D and S1E). In the parts of roots exhibiting upside-down extension, greening tissues were observed several days later. These experiments showed that co-induction of the nine TF cDNAs induced the formation of abnormal green corpulence organs in parts of the main and lateral roots. Interestingly, the newly appeared organs exhibited a negative gravitropism phenotype characteristic of shoot stems and hypocotyls. Based on phenotypic similarity to shoots, we designated these organs “shoot stem-like green organs” (SSOs). During induction, SSO formation appeared to occur at the newly generated organs from the root apical and lateral meristems (root meristems [RMs]) around the root caps. To determine which of nine candidate genes was essential for SSO formation, we prepared various constructs combining each gene and introduced them into Arabidopsis. Expression of the introduced genes was then induced in the transgenic plants (Supplemental Information and Figures S2A–S2D, Table S3). We found that simultaneous induction of two TFs, ATHB25 (*At5g65410*) (Bueso et al., 2014) and REM7 (*At3g18960*) (Mantegazza et al., 2014), led to SSO formation in Arabidopsis roots (Figure 1).

SSOs formed in the proximal regions of each lateral root and on the nascent part of the main root (in this text we represent the status of gene induction with “-ind” after the gene name, such as “ATHB25/REM7-ind”) only when both ATHB25 and REM7 were simultaneously induced (Figures 1C, 1G, and 1H: Figures S3–S5). The SSOs lacked root hairs in the epidermis. The width of SSO was more than twice the root of control plants (Figure S5B). ATHB25/REM7-ind plants exhibited a negative gravitropism response in the parts of roots, in which SSOs were generated (Figures 1G and 1H: Figures S3 and S4). ATHB25/REM7-ind plants also exhibited slight dwarfism with anthocyanin accumulation in the shoots (Figure S3, and S5C–S5F). In contrast, single-gene induction of either *ATHB25* or *REM7* (ATHB25-ind or REM7-ind, respectively) caused no obvious alteration in the visible phenotypes (Figures 1D–1F: Figures S5A and S5C–S5F). Thus, these results indicate that both ATHB25 and REM7 are sufficient to induce SSO formation in roots.

The ATHB25/REM7 plants that once formed SSOs restored normal roots in the consecutive and adjacent parts of the SSOs (Figure 1). The restoration of root growth appeared 3 days after the induction (Figure S4). The aberrant gravitropism in the root tips was observed within 3–4 days after the chemical induction, and the root hairs that indicate normal root growth increased at the same time in the adjacent parts of the immature SSOs displaying abnormal gravitropism (Figure 5A: Figure S4). The upside of the quiescent center (QC) in the root cap exhibited subtle hypertrophy (Figure S4D). This result shows that the normal root formation happens immediately after the SSO formation.

### Chlorophyll Content in Roots and the Cytokinin Effects on the SSOs

**Figure 2:**
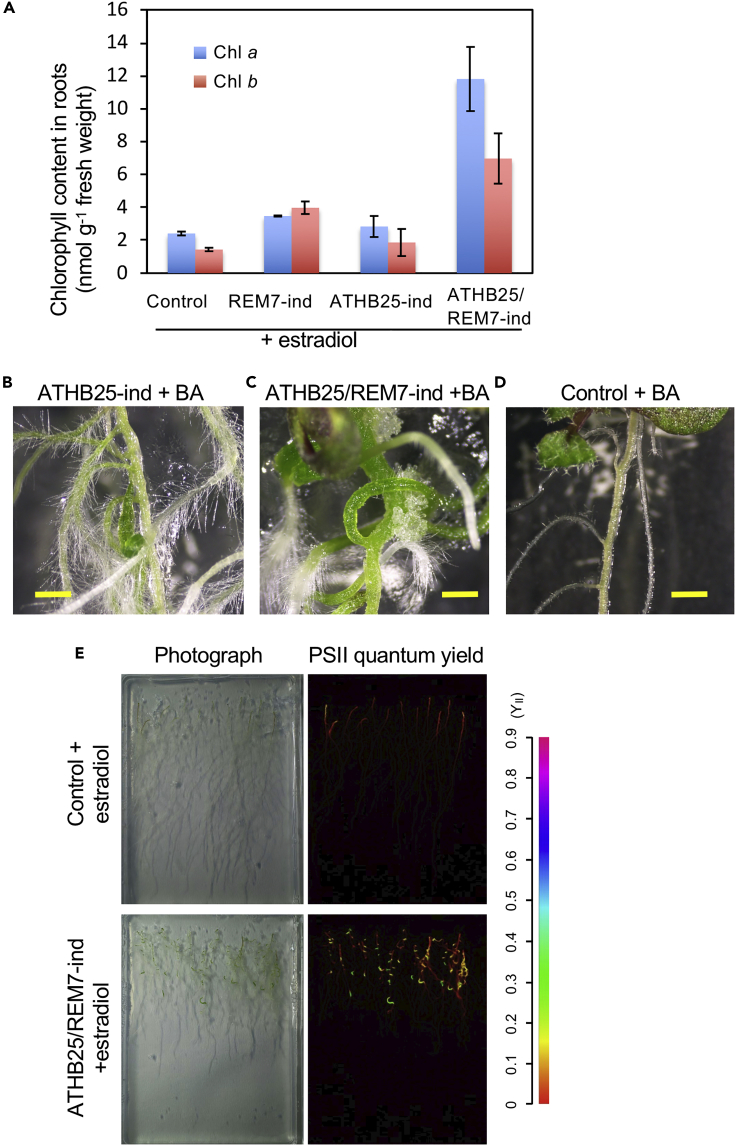
Chlorophyll Content in Roots and the Cytokinin Effects on the SSOs (A) Chlorophyll content in roots. Data are represented as mean ± SEM. (B–D) Cytokinin (BA) enhances greening in the roots of ATHB25-ind (B), ATHB25/REM7-ind (C), and control plants (D) in 12 days after the induction. Scale bars are 1 mm (B–D). (E) Chlorophyll fluorescence kinetics in the roots of control (upper panel) and ATHB25/REM7-ind plants (lower panel). The bright-field image (left panels) and PSII quantum yields (right panels) are shown. The color scale is shown to the right of the panels. See also Figures S6 and S7.

Characterization of color pigments indicated that the greening SSOs contained chlorophyll (both *a* and *b* types), as expected (Figure 2A). As the phytohormone cytokinin is generally known to enhance greening in plants (Kobayashi et al., 2012), we treated ATHB25-ind, REM7-ind, and ATHB25/REM7-ind plants with the cytokinin 6-benzylamino purine (BA) during induction (Figures 2B and 2D: Figures S5A, S5C–S5F, S6A, and S6B). As observed in the ATHB25/REM7-ind plants, the cytokinin also enhanced root greening in the single-TF ATHB25-ind plants (Figure 2B: Figures S6A and S6B) but not REM7-ind plants. As shown in Figure 2B, ATHB25 alone can stimulate SSO formation after cytokinin application, whereas REM7 cannot (Figures S5A, S6A, and S6B). Thus, the cytokinin application may bypass the function of REM7. However, the cytokinin application enhanced the greening of the ATHB25/REM7-ind plants to a much greater extent than that of the single-TF ATHB25-ind plants (Figures 2B and 2C: Figures S5A, S6A, and S6B). The cytokinin signals had additional effects on the greening in the ATHB25/REM7-ind, suggesting that the cytokinin still retains the common roles in chlorophyll biosynthesis (Kobayashi et al., 2012). Further analyses of the interaction between the phytohormones and ATHB25/REM7-ind will provide aspects on the chlorophyll biosynthesis. Our results suggest that the ATHB25 is a major regulator of root greening and that REM7 enhances the function of ATHB25.

The root greening phenotype encouraged us to investigate the developmental phase of plastids in the SSOs and to measure their photosynthetic activity. In pulse amplitude modulated (PAM) measurement (Kobayashi et al., 2017), SSO plastids exhibited greater efficiency of light utilization (ΦII) for a given amount of light than plastids in control roots, with a lower thermal dissipation of excess light energy (ΦNPQ) (Figures 2E and S7). The high ΦII in SSO plastids was attributed to high qP, suggesting that the PSII reaction center is in an “open” state in comparison with that of the control. The photosynthetic activity of SSO plastids was similar to that of leaves. Our results thus indicated that SSO plastids function as photosynthetic organelles.

### SSOs Develop Stem-like Vascular Structures

**Figure 3:**
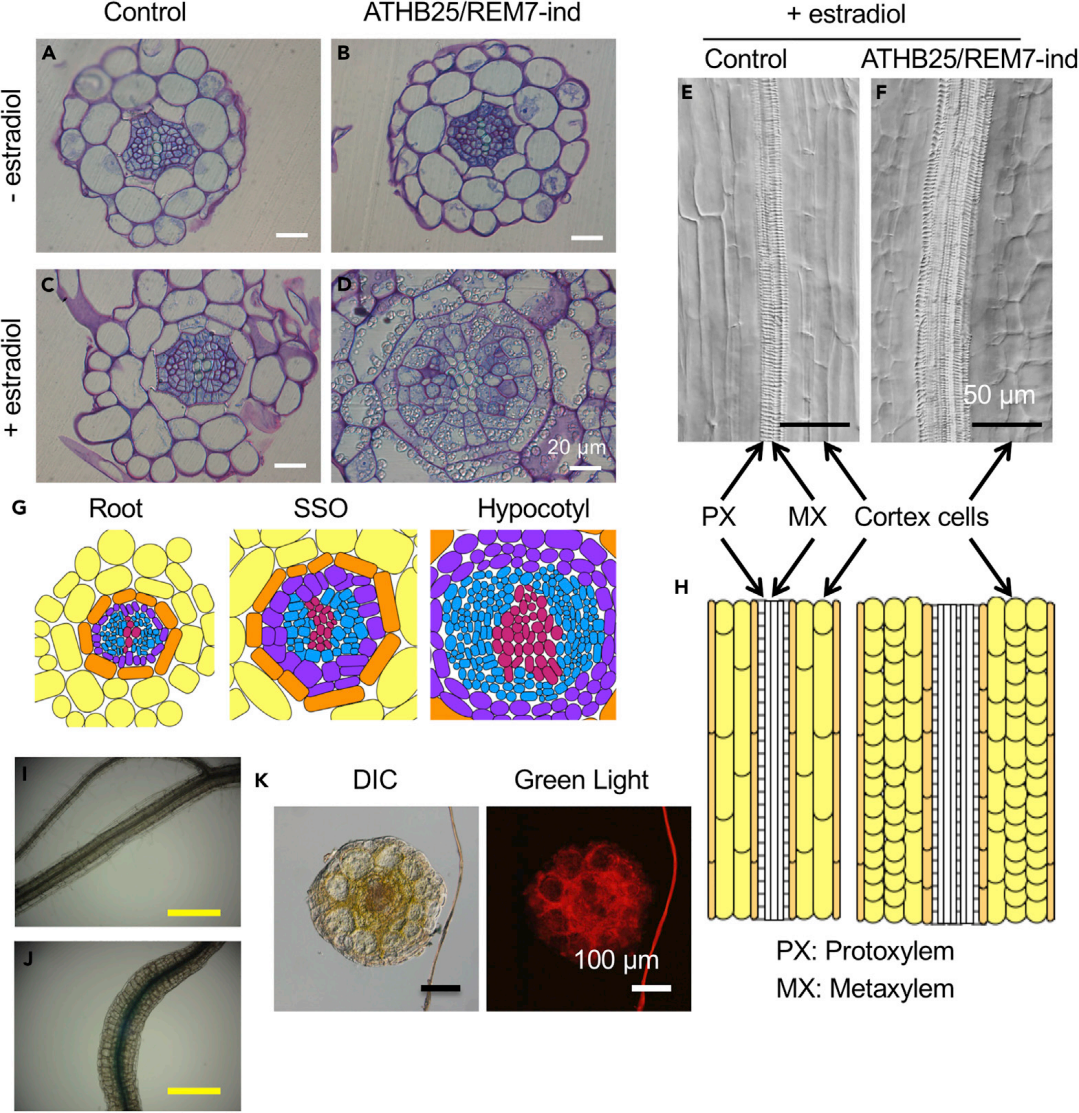
SSOs Develop Stem-like Vascular Structures (A–F) Images of sections from SSOs without (A and B) or with (C–F) estradiol treatment: cross (A–D) and vertical (E and F) sections of the root of control (A, C, and E) and ATHB25/REM7-ind plants (B, D, and F). PX, protoxylem; MX, metaxylem. (G and H) Typical schematic diagrams of the cross (G) and vertical (H) sections. The colors on the cross-sections (G) indicate xylem (pink), phloem (blue), pericycle (purple), endodermis (orange), and cortex (yellow). (I and J) WOX4:GUS expression in the root without (I) and with (J) the estradiol treatment. (K) DIC (differential interference contrast) (left) and auto-fluorescence (right) images of a cross section of an SSO indicating the distribution of chlorophyll inside the vascular tissues. Scale bars are 20 μm (A–D); 50 μm (E and F); 250 μm (I and J); and 100 μm (K). See also Figure S8.

We also investigated the structure of the vascular bundles of SSOs in the ATHB25/REM7-ind plants. Histologic assays revealed vascular enlargement and structural alterations in the SSOs (Figure 3). The number of xylem cells, particularly protoxylem cells, was higher, and the xylem cells in the vascular bundles of the SSOs were enlarged (Figures 3D, 3F, and 3G), as compared with the root of normal (control) plants (Figures 3A–3C and 3E). The enlargement of vascular bundles of the SSOs was also confirmed by observing the expansion of expression of the pro-cambium and cambium marker gene *WOX4* (Hirakawa et al., 2010) in the SSOs (Figures 3I and 3J: Figure S8B). The cortex cells of the SSOs were rounded and greater in number (Figures 3D, 3F, and 3H: Figure S8B). Interestingly, fluorescence imaging revealed chloroplasts inside the vascular bundles of SSOs (Figure 3K). Chloroplasts are not normally present in the vascular bundles of the hypocotyl or other shoot-type organs but sometimes observed in the inside of endodermis in the root (Kobayashi et al., 2012), suggesting that the SSOs partially retained root characteristics. In contrast, induction of single-TF ATHB25-ind and REM7-ind plants did not affect the vascular bundle structure (Figure S8A). Thus, both ATHB25 and REM7 are necessary for the vascular bundle phenotype of the SSOs with ectopic chloroplast development.

### Downstream Genes Regulated by ATHB25 and REM7

**Figure 4:**
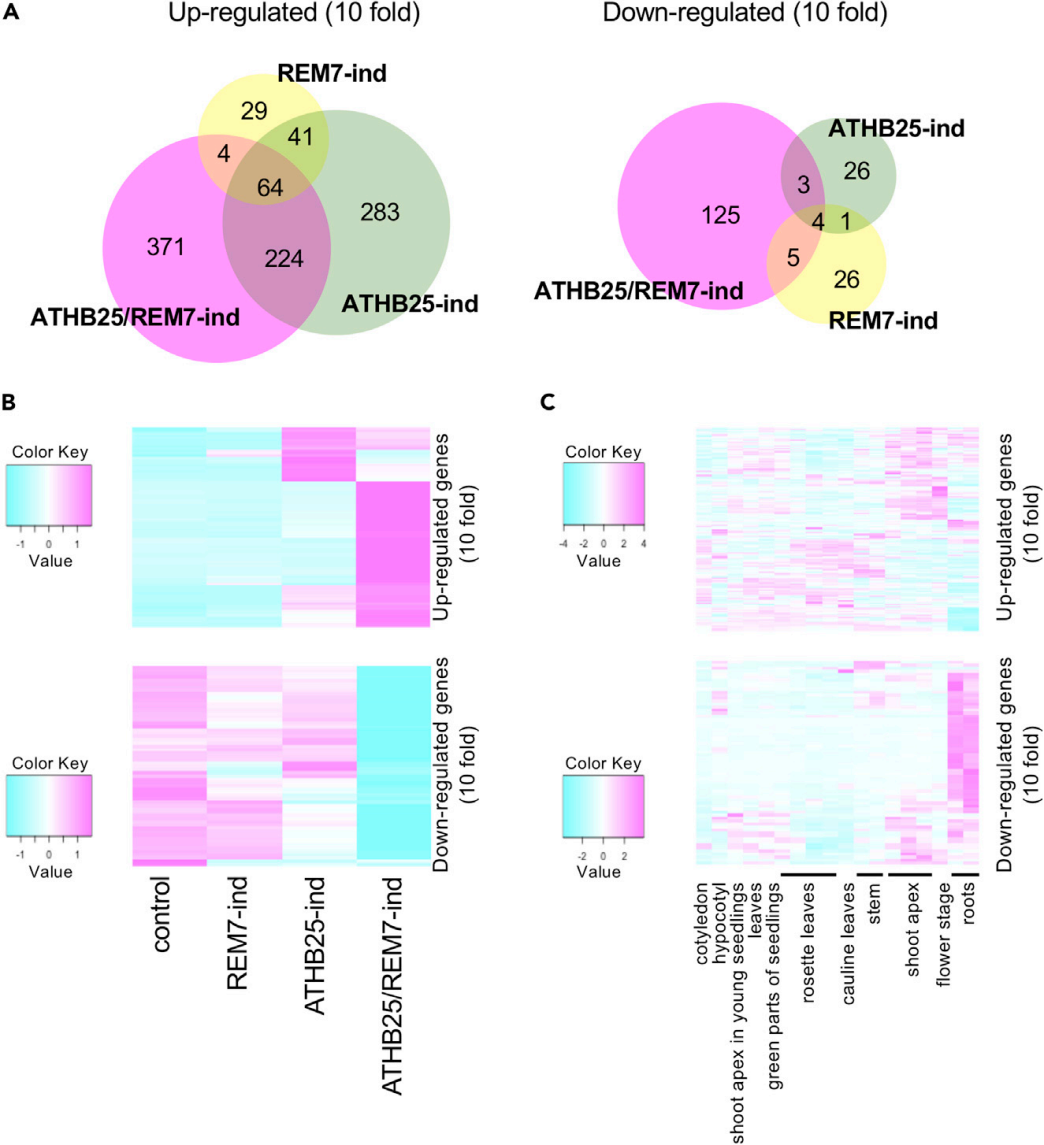
Downstream Genes Regulated by ATHB25 and REM7 (A) Numbers of 10-fold up- or down-regulated genes in ATHB25-ind, REM7-ind, and ATHB25/REM7-ind plants. (B) Expression patterns of 10-fold up- or down-regulated genes in ATHB25-ind, REM7-ind, and ATHB25/REM7-ind plants. (C) Tissue-specific expression of the genes regulated in ATHB25/REM7-ind plants. The color scale represents the expression levels. See also Figure S9.

The genes *ATHB25* and *REM7* encode a zinc-finger homeodomain protein and AP2/B3 transcription factor, respectively, and thus probably mediate transcriptional control of downstream genes. We used DNA microarrays to characterize gene expression in the SSOs by monitoring transcripts in the roots of control, ATHB25-ind, REM7-ind, and ATHB25/REM7-ind plants (Figure 4: Figure S9). In ATHB25-ind, REM7-ind, and ATHB25/REM7-ind plants, 612, 138, and 663 genes, respectively, were up-regulated at least 10-fold, and 34, 36, and 137 genes, respectively, were down-regulated at least 10-fold (Figure 4A, Data S1). A total of 371 genes up-regulated at least 10-fold and 125 genes down-regulated at least 10-fold were expressed specifically in the ATHB25/REM7-ind plants (Figures 4A and 4B). The expression patterns of the genes specifically regulated in the transgenic plants were shown in Figure 4B. Approximately 70% of the genes up-regulated at least 10-fold and more than 90% of the genes down-regulated at least 10-fold were specific in ATHB25/REM7-ind plants (Figure 4B). It is noteworthy that the low overlap between mis-regulated genes in the single and double gene inductions might depend on the indirect effects, because the samples were harvested in a week after the induction. These results thus suggest that ATHB25 and REM7 co-mediate the expression of various genes that govern SSO formation (see also the Supplemental Information, Table S4).

The genes up-regulated in the ATHB25/REM7-ind plants are expressed primarily in various shoot-derived organs in wild-type plants, whereas the down-regulated genes are expressed in the roots of wild-type plants (Figure 4C). Induction of both ATHB25 and REM7 promoted the expression of various SAM-specific genes that play critical roles in the formation and maintenance of the SAM, such as *CUP-SHAPED COTYLEDON* (*CUC*) (Aida et al., 1997), *WUSCHEL* (*WUS*) (Laux et al., 1996), *SHOOT MERISTEMLESS* (*STM*) (Endrizzi et al., 1996), and *AGAMOUS-LIKE 15* (*AGL15*) (Perry et al., 1999) (Figure S9B). These results suggest that the roots are converted to SSOs via the induction of SAM-specific genes such as *CUC* and *WUS*. The polycomb group protein *FERTILIZATION-INDEPENDENT ENDOSPERM* (*FIE*) and chromatin remodeling factor *PICKLE* (*PKL*), which maintain the transcriptionally repressed state of homeotic genes (Ogas et al., 1999; Ohad et al., 1999), were also upregulated in the SSOs, suggesting that chromatin remodeling contributes to the SSO formation. In contrast, co-induction of ATHB25 and REM7 led to repress expression of *LATERAL SUPPRESSOR* (*LAS*) (also known as *SCARECROW-LIKE 18* [*SCL18*]) (Raman et al., 2008), *LOB-DOMAIN CONTAINING PROTEIN 18* and *29* (*LBD18*, *LBD29*) (Fan et al., 2012; Xu et al., 2018), *KIP-RELATED PROTEIN 3* (*KRP3*) (also known as INHIBITOR/INTERACTOR WITH *CYCLIN-DEPENDENT KINASE INHIBITOR* [*ICK6*]) (Jun et al., 2013), and *PLETHORA1* (*PLT1*) (Santuari et al., 2016) (Figure S9B). These genes mediate the initiation of axillary meristems and lateral root formation as well as callus induction, inhibit cell division, and establish stem cells in the quiescent center. ATHB25/REM7 induction might disrupt the maintenance and development of stem cells in apical and lateral roots that overcome repression of shoot formation in roots.

### ATHB25/REM7-ind Induces *CUC2* and *WUS* Gene Expressions

**Figure 5:**
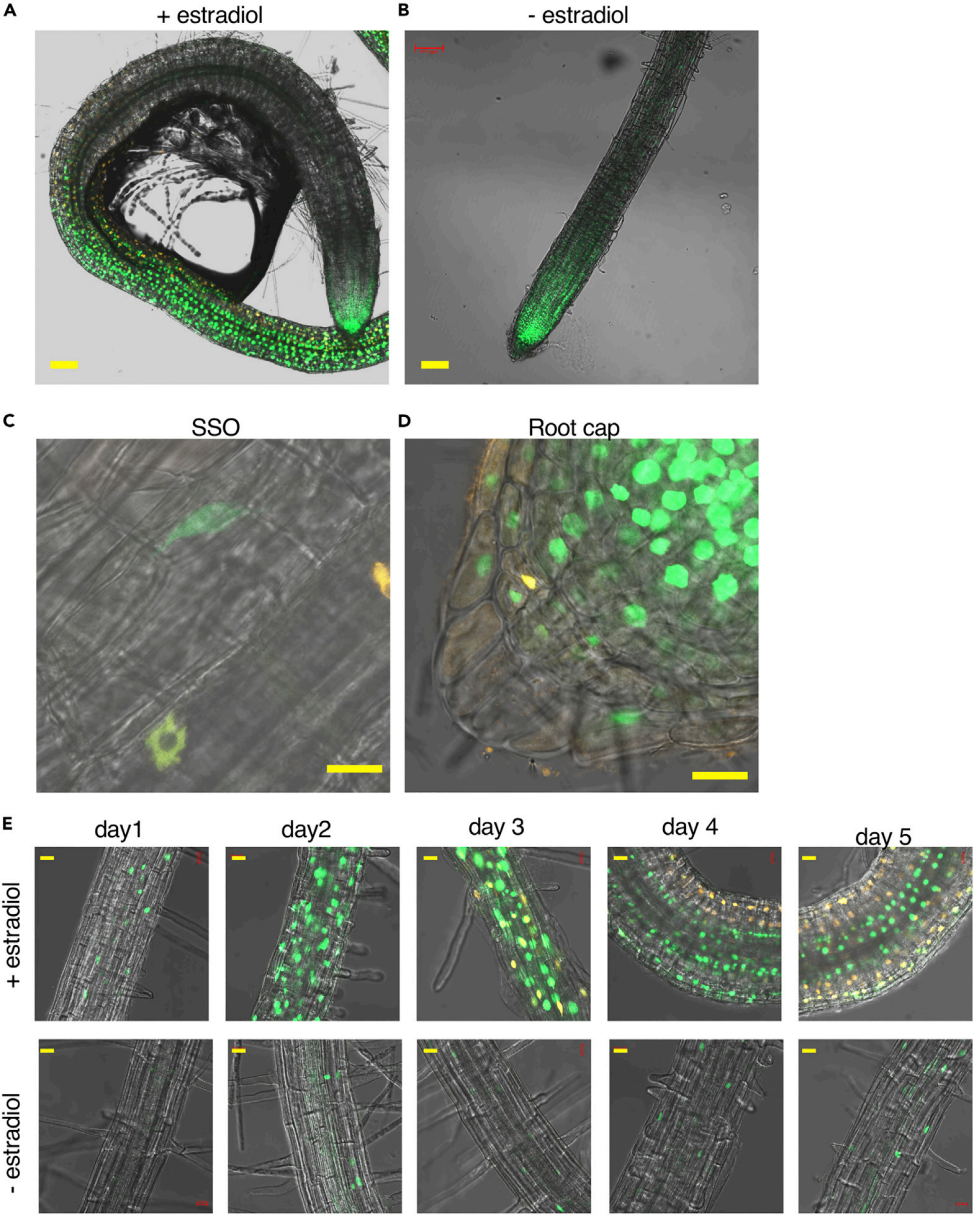
ATHB25/REM7-ind Induces *CUC2* and *WUS* Gene Expressions The *CUC2* and *WUS* gene expressions in the root of ATHB25/REM7 seedlings harboring *pCUC2*:*VENUS* (green) and *pWUS*:*dsRed* (orange) during the SSO induction with or without the estradiol. The overlap in both gene expression (yellow). (A and B) Microscopic images in 4 days after the estradiol treatment (A) and the control without the estradiol (B). (C and D) Microscopic images of the nascent region (SSOs) (C) and root cap (D) in 5 days after the estradiol treatment. (E) Time-series images during the SSO induction. Scale bars: 100 μm (A and B) and 20 μm (C–E). See also Figures S10–S12.

To investigate spatiotemporal expression of the up-regulated genes *CUC* and *WUS*, we introduced the *pCUC2*:*VENUS* (Heisler et al., 2005) and *pWUS*:*dsRed* (Reddy and Meyerowitz, 2005) reporter genes into the ATHB25/REM7-ind plants and observed these gene expressions spatiotemporally (Figure 5: Figure S10). The expression of *CUC2* gene, which is required for embryonic apical meristem formation, was observed in the whole roots especially in the nascent regions (elongation zone) generated from the RMs after the estradiol induction (Figure 5A: Figure S10), in addition to the regions around the apical meristems of shoot and main and lateral roots, where the *CUC2* expression was normally expressed (Figure 5B: Figure S10) (Smit et al., 2020). The *WUS* expression was observed in nascent regions and root cap on day 3 and later after the induction (Figures 5C–5E: Figures S10 and S11). Previously it has been reported that the cytokinin following the auxin treatment is also known to generate shoot-like organs in the roots (Rosspopoff et al., 2017). During this pre-existing root-to-shoot conversion, the expressions of *CUC2* and *WUS* genes were induced in the lateral root primordia (LRP) (Figure S12). Although the nascent pattern of *CUC2* gene expression was similar in both root-to-shoot conversions, the *CUC2* and *WUS* expression during the SSO formation displayed more broad patterns, in comparison with the reprogramming with phytohormones that activated both *CUC2* and *WUS* genes in the LRP (Figure 5). The expressions of *CUC* and *WUS* partially, but not always, overlapped during the SSO formation (Figure 5C: Figure S11). In the root cap, the *WUS* is expressed specifically at columella and lateral root cap, whereas *CUC* is at vascular and the root cap (Figure 5D: Figure S11). The expression patterns of the *WUS* differed from those induced by cytokinin (Figure S12) (Rosspopoff et al., 2017). These results show that the expressions of *CUC2* and *WUS* genes are ectopic in the SSO. This disorder of the *CUC2* and *WUS* expression might relate to the unusual localization of chloroplasts in the vascular bundles of the SSO region, which are not normally present in the vascular bundles of wild-type Arabidopsis shoot-type organs.

### Constitutive Overexpression ATHB25/REM7-ox Has Unseparated Cotyledons and Restored Roots with Aberrant Gravitropism

**Figure 6:**
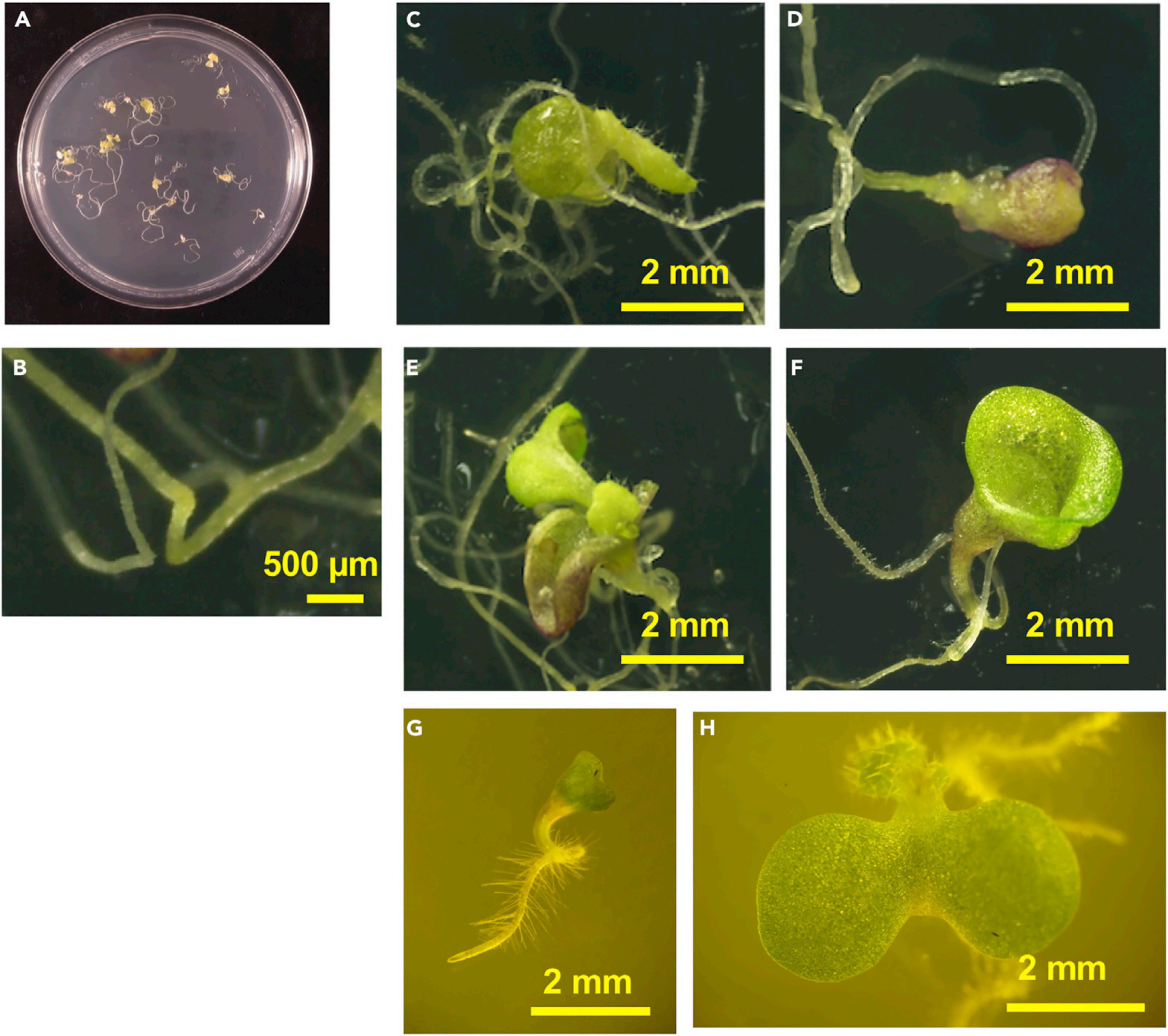
Constitutive Overexpression ATHB25/REM7-ox Has Unseparated Cotyledons and Restored Roots with Aberrant Gravitropism (A–H) The ATHB25/REM7-ox F1 plant. (A) The ATHB25/REM7-ox F1 plants were germinated on the 9-cm plates. (B) The root of the ATHB25/REM-ox F1 plants. (C–H) Phenotypic variation of the ATHB25/REM-ox F1 plants. Scale bars are 0.5 mm (B); 2 mm (C–H). See also Figure S13.

We generated plants that constitutively overproduced ATHB25 or REM7 (ATHB25-ox and REM7-ox, respectively). Neither single ATHB25- or REM7-overexpressing plants displayed visible aberrant phenotypes (data not shown). However, when we crossed ATHB25-ox with REM7-ox, some of F1 progeny (ATHB25/REM7-ox) influenced ordinal shoot formation during the early stages of development (Figure 6). After germination of the progeny seeds, the ATHB25/REM7-ox cotyledons were unseparated (Figures 6C–6H). Following the initial stage, the ATHB25/REM7-ind plants developed unhealthy shoots, grew poorly, and died, even though some plants exhibited dwarf leaves (Figures 6C, 6E, and 6H), indicating that the SAM is not completely damaged to prevent from leaf development. The ATHB25/REM7-ox exhibited aberrant gravitropism and subtle greening, followed by normal root development (Figures 6B–6G). These results confirm that the restoration of root after the SSO formation induced by the estradiol method is not the result of a depletion of estradiol during plant growth but is a developmental process.

The transfer DNA (T-DNA) tagged lines of ATHB25 or REM7 and the double mutants prepared from the T-DNA tagged lines exhibited no obvious changes in phenotypes (see Supplemental Information and Figure S13). As both *ATHB25* and *REM7* genes are members of the ZINC FINGER HOMEODOMAIN (ZHD) and REM (REPRODUCTIVE MERISTEM) families, respectively, some of these paralogs may mask the phenotypes of *athb25*/*rem7* double mutants.

In addition to the loss- and gain-of-function experiments, we carried out GUS reporter assays of these genes (see Supplemental Information and Figure S13). *ATHB25* was not only expressed around the SAMs but also at the border and boundary domains between organs, where the vascular cells divided, and *REM7* was expressed in the veins of leaves around the SAM of seedlings and mature plants (Figure S13). We also observed no physical interaction between the ATHB25 and REM7 proteins based on yeast two-hybrid assays (Figure S13). These data suggest that the TFs ATHB25 and REM7 are part of a complex process in which the factors localize separately in distinct cells or paralogs localize closely in the same cells or adjacent cells to mediate shoot stem development.

## Discussion

Here we demonstrated the artificial formation of SSOs in roots by simultaneous induction of two TFs, ATHB25 and REM7, in which a synthetic biological approach for gain of function was taken to find the combination of the two genes. SSOs are differentiated organs that exhibit negative gravitropism and vascular structures that are unique in shoot stems and hypocotyls but not in roots (Figures 1 and 3). The SSOs have chloroplasts exhibiting photosynthetic activity similar to that of leaves (Figures 1 and 2). Co-induction of ATHB25 and REM7 induces the expression of shoot-specific genes but suppresses that of root-specific genes (Figure 4). These results indicate that the simultaneous induction of these TFs mimics major shoot stem characteristics in the roots. Intriguingly, ATHB25/REM7-ind plants that once formed the SSOs restore normal root growth in the consecutive and adjacent parts of the SSOs even under the conditions of the ATHB25/REM7 induction (Figures 1 and 5). The restoration of root development is also observed in the ATHB25/REM7-ox that expressed the TFs constitutively (Figure 6). Thus, this synthetic biological approach induces shoot stem characteristics in the root without dedifferentiation and subsequently restores root growth.

Our results suggest that ATHB25 and REM7 play multiple roles in the establishment of shoot stem characteristics in roots. Up-regulation of these TFs induced expression of the *CUC*, *WUS*, and *STM* genes (Figures 4 and 5), which function as fate determinants in the apical meristem (Aida et al., 1997; Endrizzi et al., 1996; Gallois et al., 2002, 2004; Laux et al., 1996; Mayer et al., 1998), and repressed the expressions of genes such as *LAS*/*SCL18*, *LBD18*/*29*, *KRP3*/*ICK6*, and *PLT1* (Figure S9B), which are involved in the initiation of axillary meristems, establishment of stem cells in the quiescent center, lateral root formation, and negative regulation of cell division (Fan et al., 2012; Jun et al., 2013; Raman et al., 2008; Santuari et al., 2016; Xu et al., 2018). The induction of shoot stem characteristics in roots differs from the dedifferentiation that occurs when the SAM-identity gene *WUS* is ectopically expressed in roots (Gallois et al., 2002, 2004). As apparent dedifferentiation was not observed during the SSO formation period, formation of the artificial organ is likely to be induced in the somatic organs newly generated from the RM (Figures 1 and 5). The *CUC2* and *WUS* genes that play critical roles in the SAM formation were induced in the elongation zone, whereas the induction of the *WUS* expression was not detected in the RMs themselves (Figure 5: Figures S10 and S11). As the key regulator genes *CUC2* and *WUS* were induced in the ATHB25/REM7-ind, the ATHB25 and REM7 are likely to be involved in the formation of the SAM intermediates or differentiation of shoot stems in the elongation zone. Although the phytohormone cytokinin was reported to induce the conversion from the lateral root primordia (LRP) into the shoots in the previous studies (Rosspopoff et al., 2017), SSO initiation in ATHB25/REM7-ind did not require the phytohormones. Spatiotemporal patterns of the *WUS* expression in the SSO differ from those in the lateral root primordia formed by the phytohormone treatments, in which WUS is expressed at the apical stem cell (Figure 5: Figures S10–S12). Collectively, our results showed that SSO formation differs from any pre-existing reprogramming via apparent dedifferentiation or the LRP with phytohormones. We hypothesize that these TFs alter the function of the somatic cells to direct their fate toward differentiation of shoot stems.

Root development was restored after SSO formation (Figure 1), not due to the depletion of the gene inducer β-estradiol. We showed that the restoration of root growth begins at least day 3 (Figure S4), although the induction of GUS gene on the estradiol plate was kept for 12 days (data not shown). The ATHB25/REM7-ox also exhibited the negative gravitropism and subtle greening in the adjacent zone of the hypocotyl and normal growth (Figure 6), confirming that the restoration of root growth is a developmental process even under the action of the ATHB25/REM7. The elongation zone is converted to the organs with shoot stem characteristics by ectopic induction of the two TF genes, and the developed organs might restore the root development. In contrast to the previous reports in that the RMs were converted into the organs with shoot characteristics (Gallois et al., 2004; Ikeda et al., 2009; Ikeuchi et al., 2016; Rosspopoff et al., 2017), the RM state is retained in the root cap during the SSO formation. The two TFs seem to induce shoot stem characteristics directly from the somatic cells rather than from the SAMs that were converted from the RMs. Our hypothesis is that cells with shoot stem characteristics are generated in the elongation zone adjacent to the quiescent center cell of the RM (Figure 5), and the organs with shoot stem characteristics induced the activities of RMs adjacent to the SSOs. Future study is needed to understand the molecular mechanisms underlying the SSO formation.

It is noteworthy that the *WUS* expression is found in the SSOs (Figure 5), although it is confined in the SAM in the wild-type (Heisler et al., 2005), and the expressions of *WUS* and *CUC* are not always overlapped in the SSO (Figure 5). Dedifferentiation occurs when the *WUS* gene is ectopically expressed in roots (Gallois et al., 2004; Ikeda et al., 2009), but no dedifferentiation is apparent when the *WUS* expression is induced in the SSOs. The inconsistency might be explained by the lack of ectopic expression of the *WUS* in the meristematic cells even when the ATHB25/REM7 is activated, as the *WUS* functions in meristems (Gallois et al., 2004). It was also inconsistent with the previous reports describing that overexpression or ectopic induction of the *CUC* genes (*CUC*-ox) deepened serration of cotyledon and leaf margins but did not exhibit the *cuc*-like phenotypes (Li et al., 2020; Nikovics et al., 2006; Takada et al., 2001), that the ATHB25/REM7-ox F1 plants display the unseparated cotyledon as seen in the phenotype of the *cuc* mutant (Aida et al., 1997). It seems that the ATHB25/REM7 acts not only on the expression of the *CUC* genes and subsequent expression of *WUS* but also on an unknown function that works to maintain the SAM properly. Although microarray data showed that the STM was induced at the later stage of SSO formation in the ATHB25/REM7-ind, spatiotemporal induction of STM gene was not observed in the SSOs within 5 days after the induction (data not shown). These results suggest that the ATHB25/REM7 action is sufficient to induce the SSOs from the RMs but not enough to generate the SAM identity, by which the polarity of *WUS* and *CUC* expression is disturbed in the SSOs.

Although we did not conduct further analyses of inherent functions of ATHB25 and REM7 in apical meristems, our study of T-DNA tag lines, yeast two-hybrid assays, and GUS reporter assays of these genes provides data regarding their functions and will be useful in future studies on the molecular mechanisms of the establishment of shoot stem characteristics in Arabidopsis (see the Supplemental Information: Figure S13).

In conclusion, although recent studies of plant regeneration have revealed many aspects of the dedifferentiation processes that lead to the formation of calluses and adventitious embryos in roots (Gallois et al., 2004; Ikeuchi et al., 2015; Iwase et al., 2017; Waki et al., 2011), researches of artificial induction of shoot stem without dedifferentiation are limited to date. Our findings suggest that the TFs ATHB25 and REM7 change the fates of the elongation zone adjacent to the RMs to develop shoot stem characteristics without apparent dedifferentiation. The SSO formation seems to occur in the somatic cells but not in the RMs themselves (differentiation zone). The elongation zone without meristems may explain why ATHB25/REM7-ind induced the organs with shoot-stem characteristics instead of the SAMs, which might be converted from the root meristems (Rosspopoff et al., 2017). Intriguingly, a subsequent restoration of root growth occurs in the consecutive and adjacent parts of the SSOs even under the conditions of the gene activation. Revealing the details of the processes by which artificial organs such as SSOs develop will accelerate research aimed at fully elucidating the mechanisms of plant development and regeneration, particularly in the emerging field of synthetic biology (Benning and Sweetlove, 2016; Nemhauser and Torii, 2016).

### Limitations of the Study

The synthetic biological approach to induce the SSO in the roots by the combined action of ATHB25 and REM7 does not, of necessity, affirm that the combination works in the wild-type plant; that is the limitation of such approach. However, it suggests that such combined protein function induces the stem at the SAM in the wild-type. The present study did not provide clear evidence of the same spatiotemporal location of these gene expressions in the wild-type. As these genes have paralogous genes on the genome, future research will clarify a genuine set of genes that are involved in the stem induction.

Whether the inductions of the SAM identity genes, *CUC1*/*CUC2*, *WUS*, and *STM*, by the combined action of ATHB25 and REM7 are independent of the SSO formation in the roots or not remains to be elucidated. The inductions of the SAM identity genes in the SSO are aberrant as they, except *CUC1*/*CUC2*, are expressed strictly at the SAM but not in shoots in the wild-type plants. The present study showed no visible induction of *WUS* and *STM* near the RM or the zone of cell division at the early stage of the activation of ATHB25 and REM7, although the zone of cell division region exhibited slight corpulent cells. Analyses of the histological changes at the zone of cell division after the activation of ATHB25 and REM7 will address the question in future research.

The penetration of the chemical inducer from the surface of the roots also complicates this discussion. Further studies such as a single cell induction of these TFs will aid our understanding of this phenomenon in more detail.

### Resource Availability

#### Lead Contact

Further information and requests for resources and reagents should be direct to and will be fulfilled by the Lead Contact, Shigeru Hanano (hanano@kazusa.or.jp).

#### Materials Availability

This study did not generate new unique reagents.

#### Data and Code Availability

The nucleotide sequences of the vectors reported in this paper have been submitted to the DNA Data Bank of Japan (DDBJ) under accession numbers GenBank: LC217876 and LC217877. The microarray experiment data described in this publication have been deposited in the NCBI's Gene Expression Omnibus and is accessible through the GEO Series accession number GEO accession: GSE105401.

## Methods

All methods can be found in the accompanying Transparent Methods supplemental file.

## Supplemental Information

Document S1. Transparent Methods, Figures S1–S13, and Tables S1–S4

Data S1. List of the Target Genes Regulated by ATHB25 and REM7, Related to Figure 4 This file is provided as the EXCEL format. Individual sheets contain the gene lists of 10-fold upregulated and downregulated in ATHB25/REM7-ind, ATHB25-ind, REM7-ind. Gene Symbol, Values of fold change (FC), Regulation, Gene name, and Description are represented. The data are related to Figures 4 and S9.

## Footnotes

Supplemental Information can be found online at https://doi.org/10.1016/j.isci.2020.101332.
