# Supplementary material for: An Artificial Conversion of Roots into Organs with Shoot Stem Characteristics by Inducing Two Transcription Factors
Source: iScience. 2020 Jul 14;23(7):101332. doi: 10.1016/j.isci.2020.101332 (PMC7385925; doi:10.1016/j.isci.2020.101332)
Supplement: Document S1. Transparent Methods, Figures S1–S13, and Tables S1–S4 [file mmc1.pdf]

iScience, Volume *23*

Supplemental Information

An Artificial Conversion of Roots into Organs with Shoot Stem Characteristics by Inducing Two Transcription Factors

Shigeru Hanano, Hajime Tomatsu, Ai Ohnishi, Koichi Kobayashi, Yuki Kondo, Shigeyuki Betsuyaku, Eiji Takita, Yoshiyuki Ogata, Keishi Ozawa, Kunihiro Suda, Tsutomu Hosouchi, Takahiro Nagase, Hideyuki Suzuki, Nozomu Sakurai, Hiroshi Masumoto, Hiroo Fukuda, and Daisuke Shibata

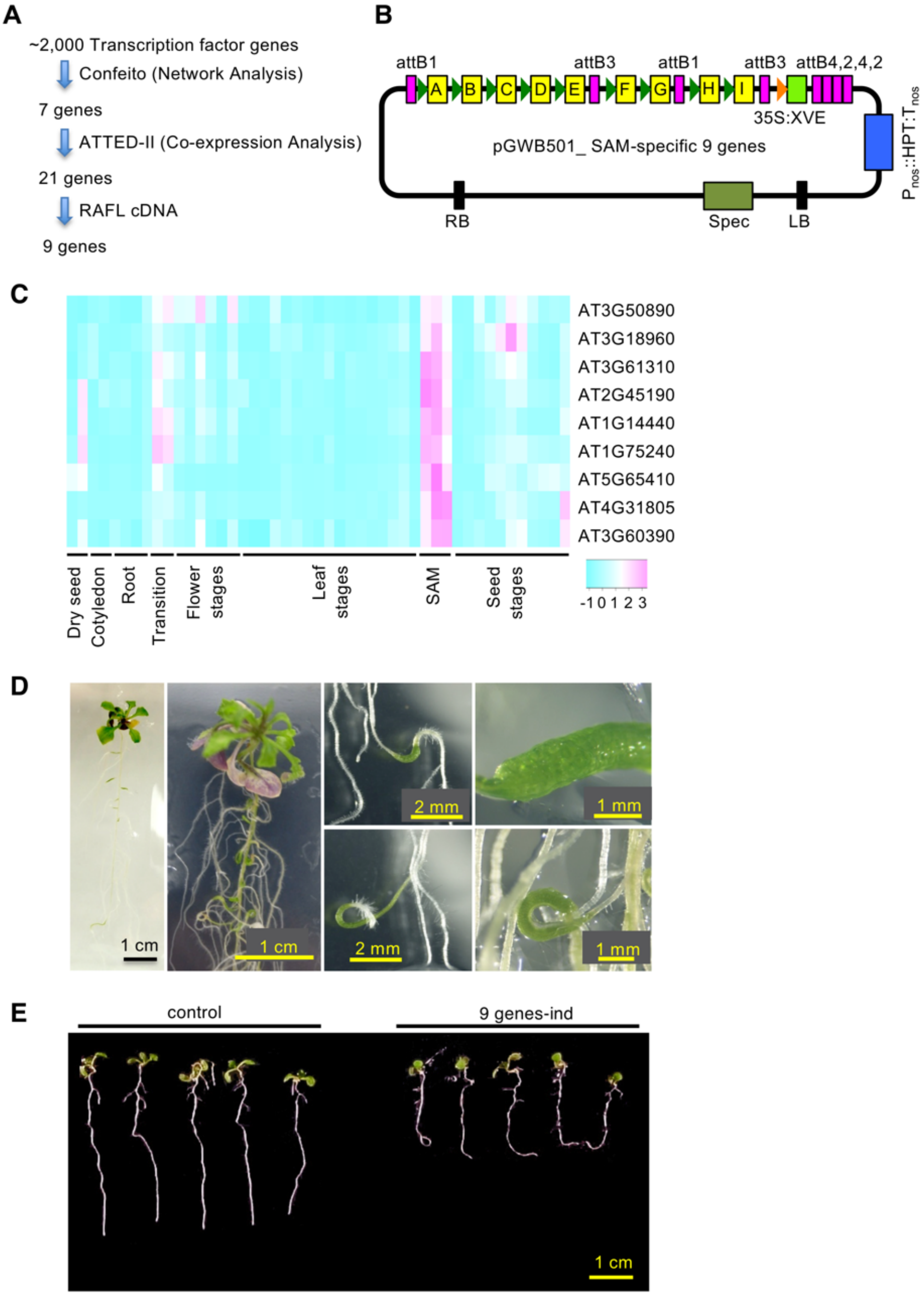

Figure S1. Selection and cloning of nine genes encoding the SAM-specific TFs, Related to Figure 1. (A) The selection scheme for the SAM-specific TFs. (B) The plasmid for the SAM-specific-gene induction is represented. These cloned cDNAs are represented as yellow boxes with the following letters, A to I (Table S1). Three sets of ligated cassettes, set A, set B and set C (Table S1: Figure S2C), were cloned into *att*B sites (pink boxes). Each cDNA was driven by a Lex A operator (green triangles) and was assumed to be inducible with estradiol treatment. (C) The tissue-specific expression of the genes used for the constructs is shown as a heat-map. The map was drawn based on the public array data for Arabidopsis development (accession no. E-TABM-17). (D) Phenotypes of the plants co-expressing nine cDNAs that encode SAM-specific TFs. The transgenic plants were treated with 5 µM 17-ß-estradiol for three weeks. (E) The gravitropism of the transgenic plants expressing nine cDNAs under XVE-operator control; control (left) and transgenic plants (right: 9 genes-ind). These plants were treated with 1 µM 17-ß-estradiol for four days. Scale bar = 1 cm.

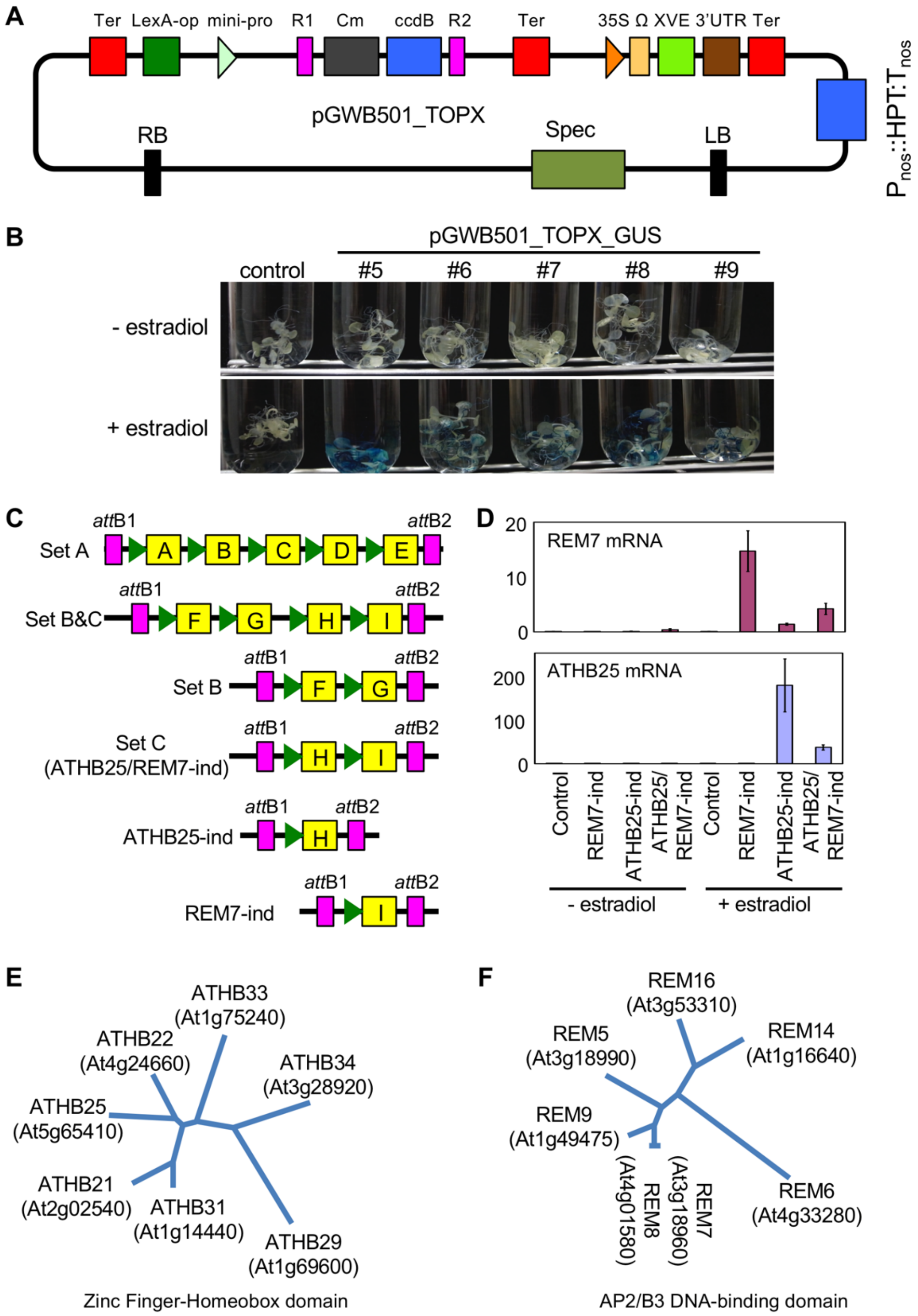

Figure S2. Construction of the chemically inducible vector and chemical induction of ATHB25 and REM7, Related to Figure 1. (A) The estradiol-inducible Gateway vector, pGWB501_TOPX constructed in this study. (B) Estradiol induction using the pGWB501_TOPX vector. (C) Constructs for the evaluation of the nine gene combination. Each set A, set B and set C were cloned in the PRESSO method and then ligated in the Multiple Gateway Cloning (Table S1: Figure S1B). (D) ATHB25 and REM7 gene expressions were confirmed in the inducible transgenic plants (REM7-ind, ATHB25-ind, and ATHB25/REM7-ind). Here, we show the relative values of expression of ATHB25 and REM7 from a typical line. Data are represented as mean +/- SEM. (E and F) Phylogenetic tree of ATHB25 (E) and REM7 (F).

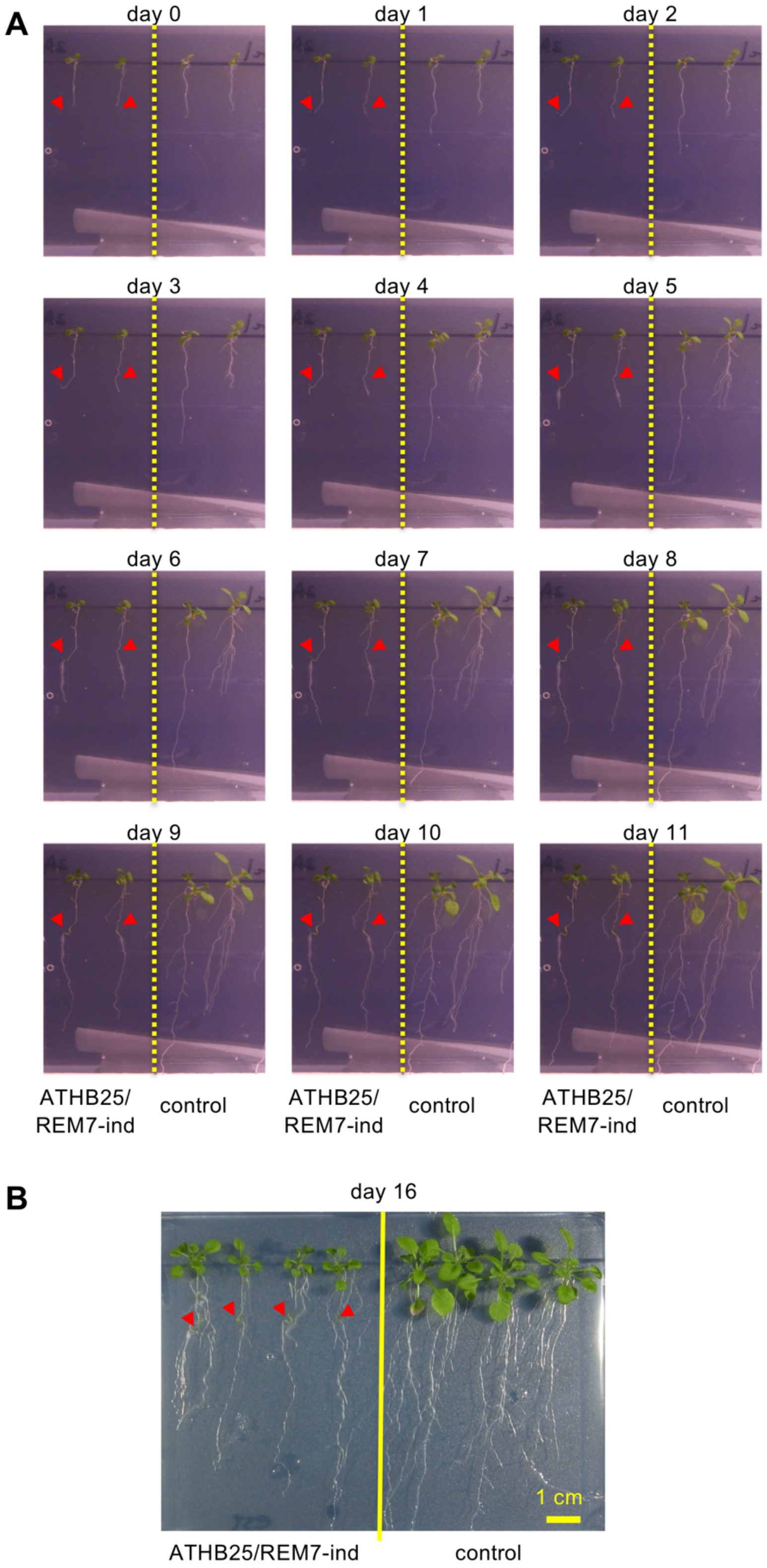

Figure S3. Real-time images of the SSO formation by co-induction of ATHB25 and REM7, Related to Figure 1. Time-series of photograph during the SSO formation between 0 and 11 days (A), and in 16 days (B) after the estradiol treatment. (A) Each 2 plants of ATHB25/REM7-ind (left) and control (right) are represented from central parts of image (B). Red arrows indicate the regions for the SSO formation.

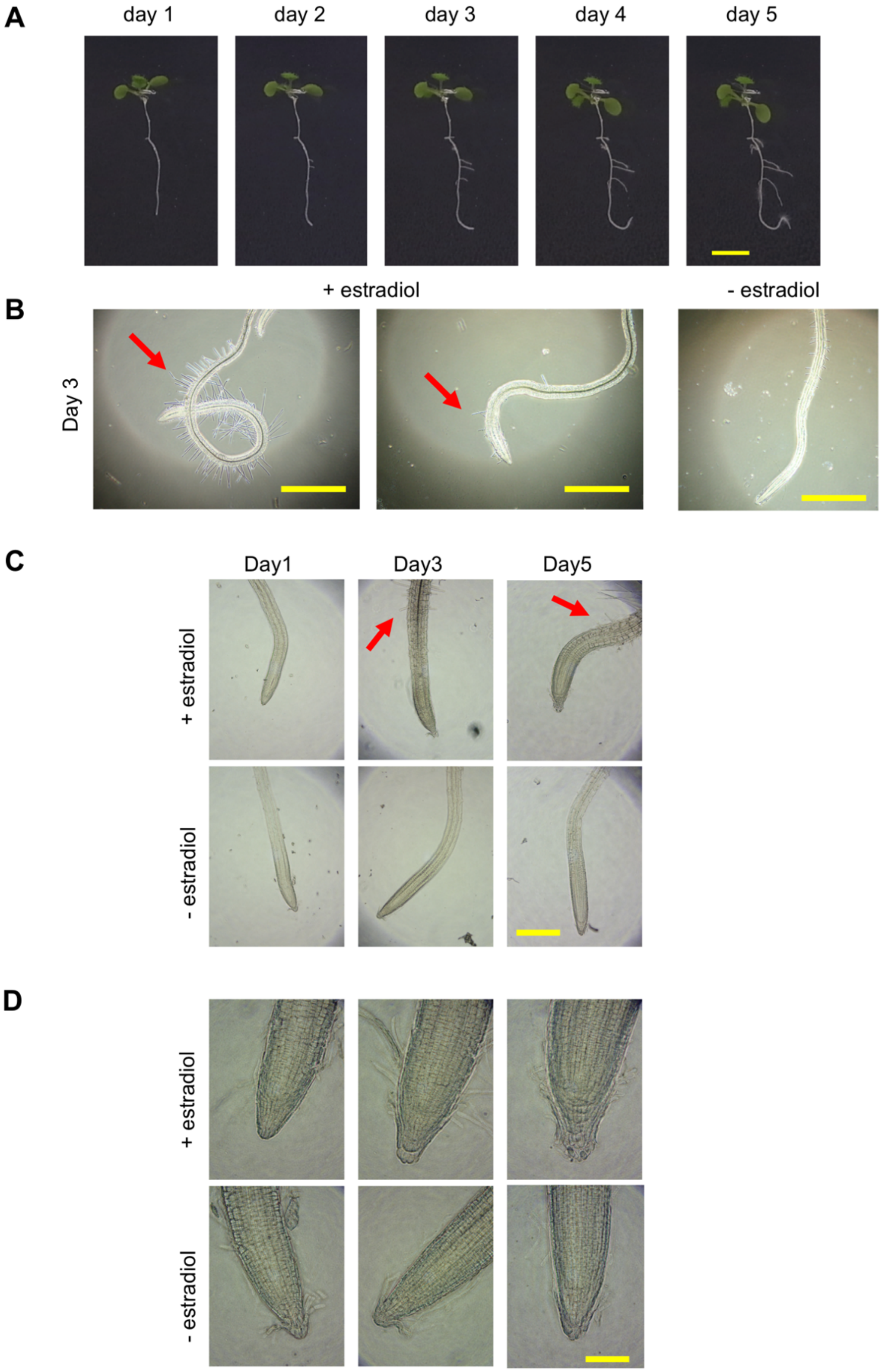

Figure S4. Real-time images of the SSO formation on the early stage of the SSO formation, Related to Figure 1. (A) Details of plant growth between day 1 and day 5 of SSO formation. (B) Root growth in day 3 of SSO formation with or without estradiol treatment. (C, D) Details of root tip growth between day 1 and day 5 of SSO formation. Scale bar = 5 mm (A), 1 mm (B), 400 µm (C) and 100 µm (D).

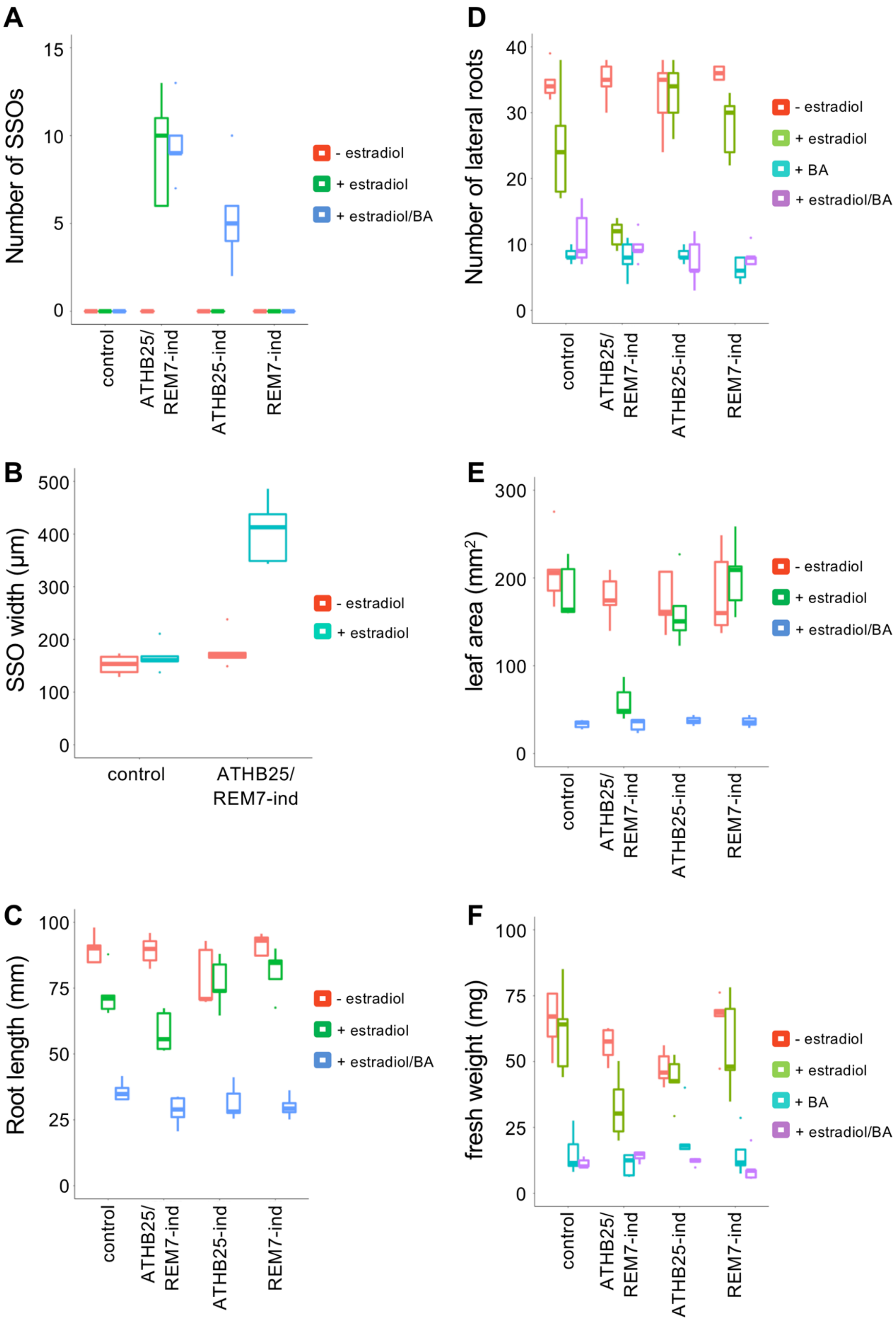

Figure S5. Phenotypes of the ATHB25/REM7-ind plants, Related to Figure 1. (A) Number of SSOs: (B) SSO width: (C) Root length: (D) Number of lateral roots: (E) leaf area (F) fresh weight. Data are represented as mean with first and third quartile and 95% confidence interval of median.

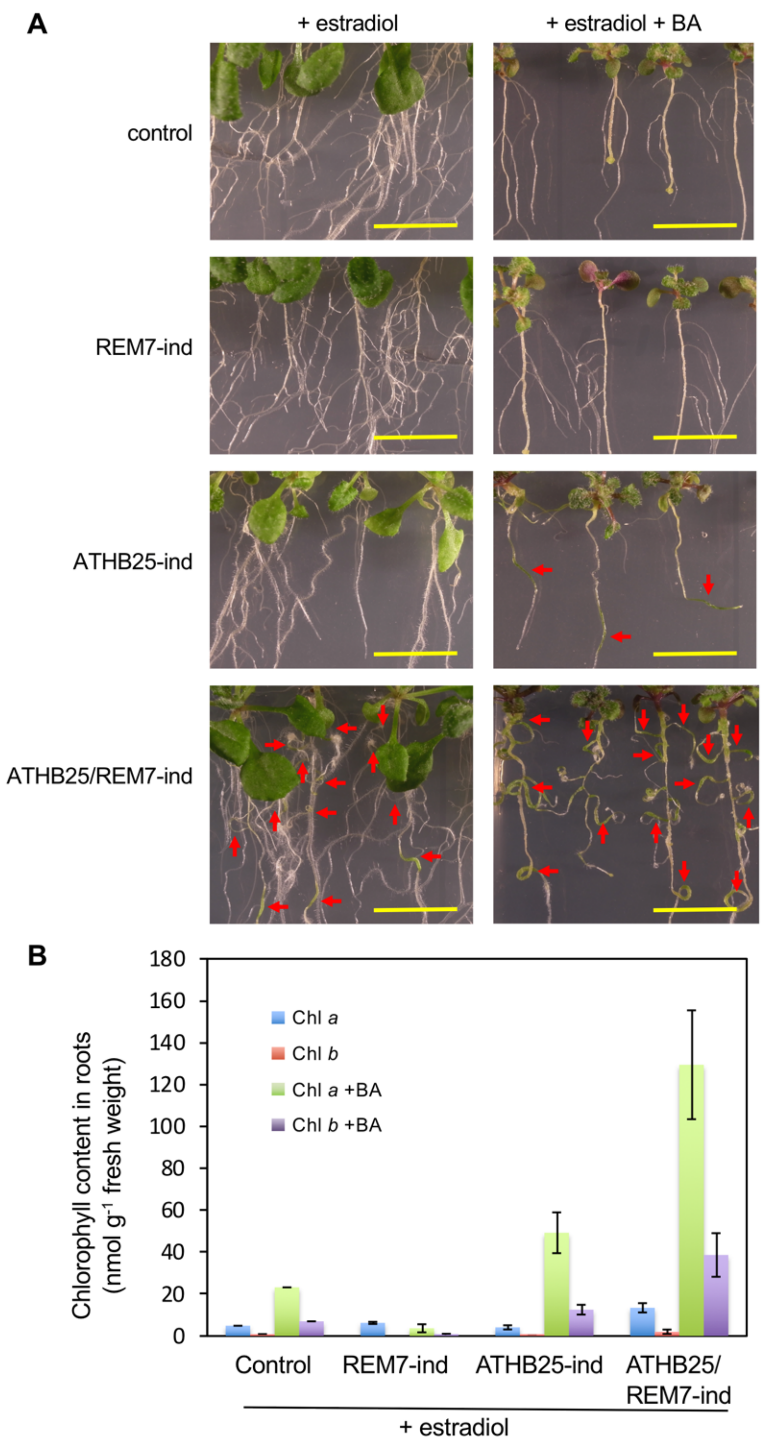

Figure S6. Chemical induction of ATHB25 and REM7 with a cytokinin, Related to Figure 2. (A) Seven-day-old seedlings of plants harboring ATHB25-ind, REM7-ind, or ATHB25/REM7-ind were grown on 1/2 MS-1% sucrose plates containing vitamins and 5μM estradiol with (+BA) or without 1 μM of the cytokinin, 6-benzylamino purine (BA) for 10 days. The red arrows indicated SSOs. Without the cytokinin (left panels), the SSOs were observed only in the roots of the ATHB25/REM7-ind plants (as shown in Figure 1 C, G, and H). In contrast, ATHB25-ind generated the SSOs in the roots with the addition of cytokinin (right panels). The cytokinin also enhanced SSO formation in the ATHB25/REM7-ind plants. Scale bars: 1 cm. (B) Chlorophyll contents in the roots of the ATHB25-ind, REM7-ind, and ATHB25/REM7-ind plants with or without cytokinin. Data are represented as mean +/- SEM.

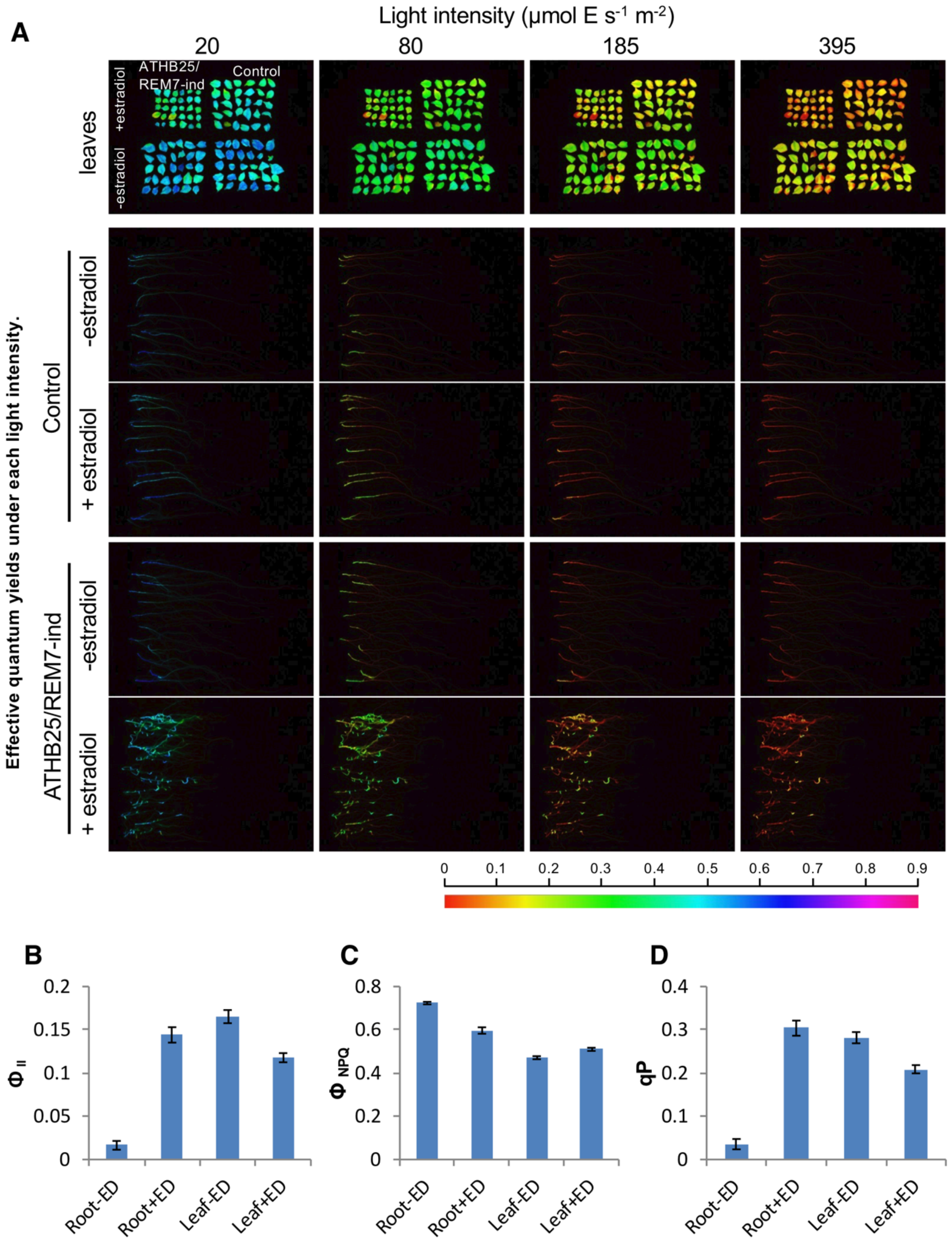

Figure S7. Photosynthetic activity in the transgenic plants, Related to Figure 2. Photosynthetic activity was measured with the IMAGING-PAM fluorometer (WALZ). (A) Effective quantum yields under each light intensity. The leaf images indicated that the photosynthetic activity between the control and ATHB25/REM7-ind plants did not change significantly but simultaneous induction of ATHB25 and REM7 reduced leaf size. In the root image, high photosynthetic activity was observed at the SSOs in the ATHB25/REM7-ind plants. (B-D) Quantitative data from the IMAGING-PAM fluorometer: (B) Φ(II), (C) Φ(NPQ) and (D) qP. ED: estradiol. Error bar is S. E. (n = 5).

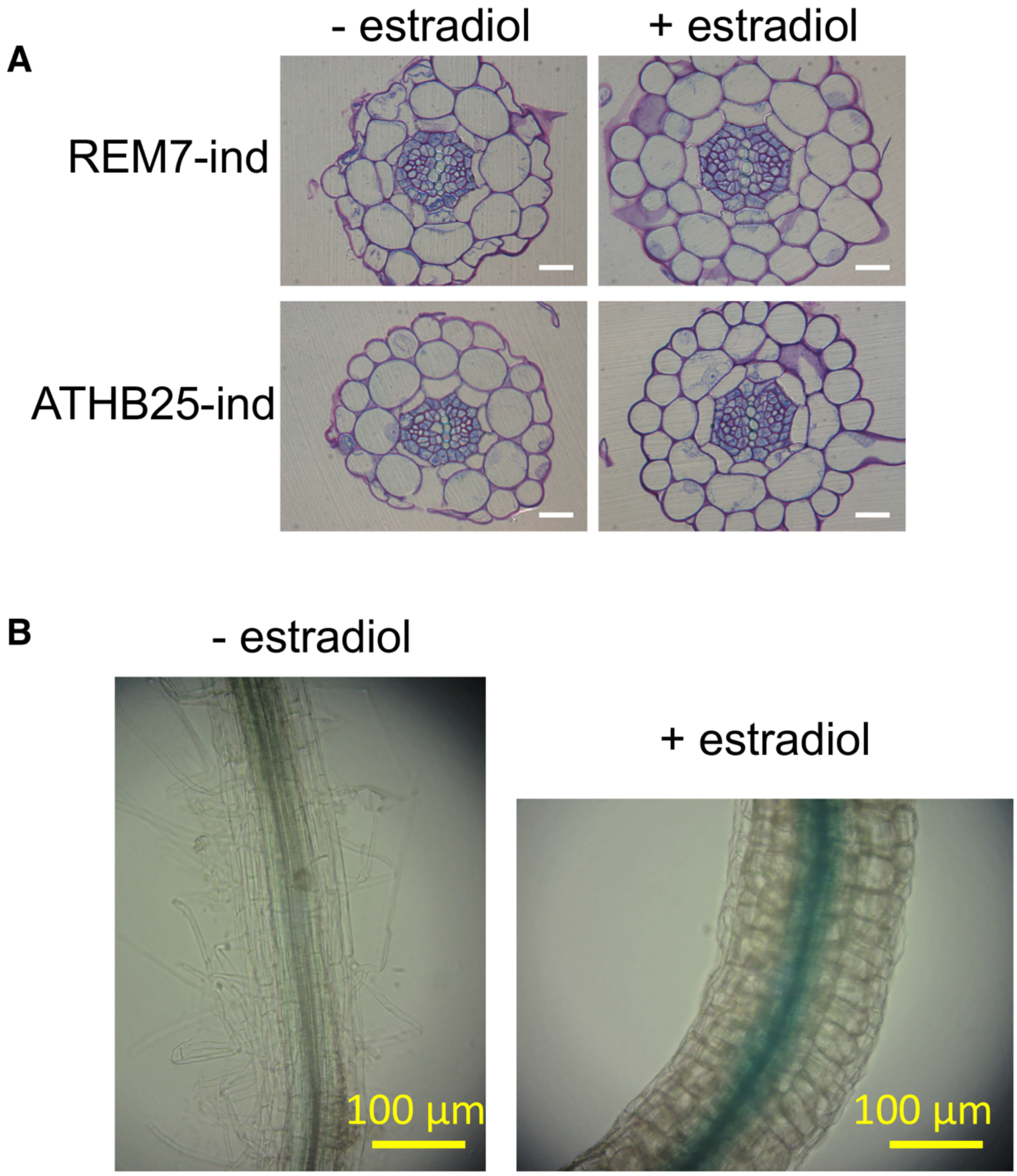

Figure S8. Cross sections of the root, Related to Figure 3. (A) The roots of the ATHB25-ind and REM7-ind plants with or without 5 µM estradiol were sectioned and stained as described in the METHOD DETAILS. (B) WOX4:GUS expression in the ATHB25/REM7-ind root with or without the estradiol treatment.

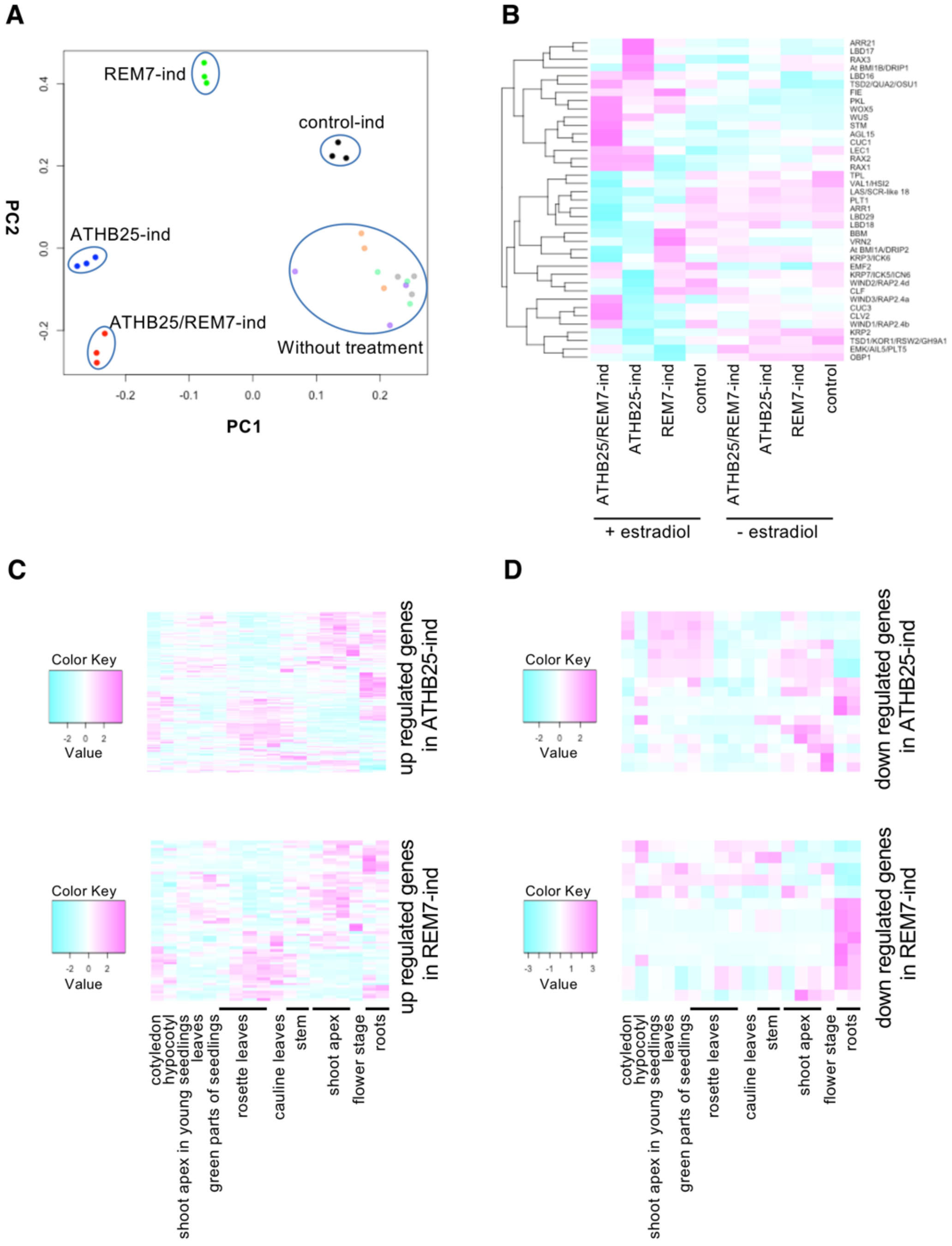

Figure S9. The target genes regulated by ATHB25 and REM7, Related to Figure 4. (A) A principal component analysis (PCA) of the microarray experiments. Red, ATHB25/REM7-ind; Blue, ATHB25-ind; Green, REM7-ind, and Black, control plants. The pale color shows data from samples without estradiol induction. (B) Expression of genes involved in embryogenesis and meristem development. (C) The expression map of up-regulated genes during plant development by individual induction of ATHB25 and REM7. (D) The expression map of down-regulated genes during plant development by individual induction of ATHB25 and REM7. In these heat-maps of the ATHB25- or REM7-regulated genes, some of the up-regulated genes were expressed in the shoots, while some of the down-regulated genes were expressed in the roots. Many of the down-regulated genes in the REM7-ind plants are known as root-specific genes. However, in comparison with the data from the ATHB25/REM7-ind plants (Figure 4C), the effects of the single ATHB25 and REM7 induction seem to be limited.

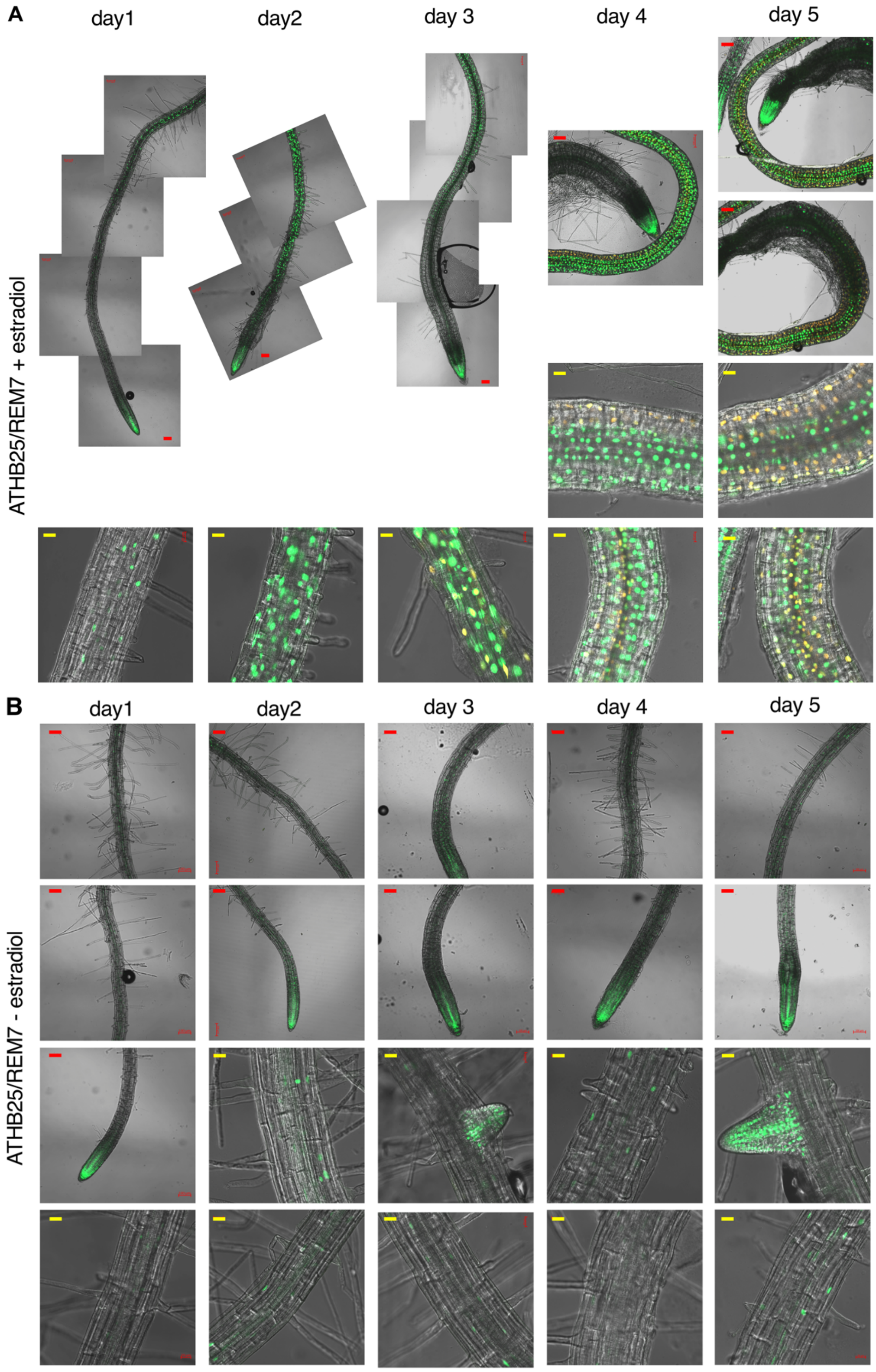

Figure S10. Expression of *CUC2* and *WUS* genes during SSO induction, Related to Figure 5. The *CUC2* and *WUS* gene expressions in the root of ATHB25/REM7 F1 seedlings harboring *pCUC2*:*VENUS* (Green) and *pWUS*:*dsRED* (Orange) during the SSO induction with (A) or without the estradiol treatment (B). The overlap of *CUC2* and *WUS* expression was represented as yellow. Some figures are selected and used as duplicated in Figure 5. Scale bars: 20 µm (yellow), 100 µm (red).

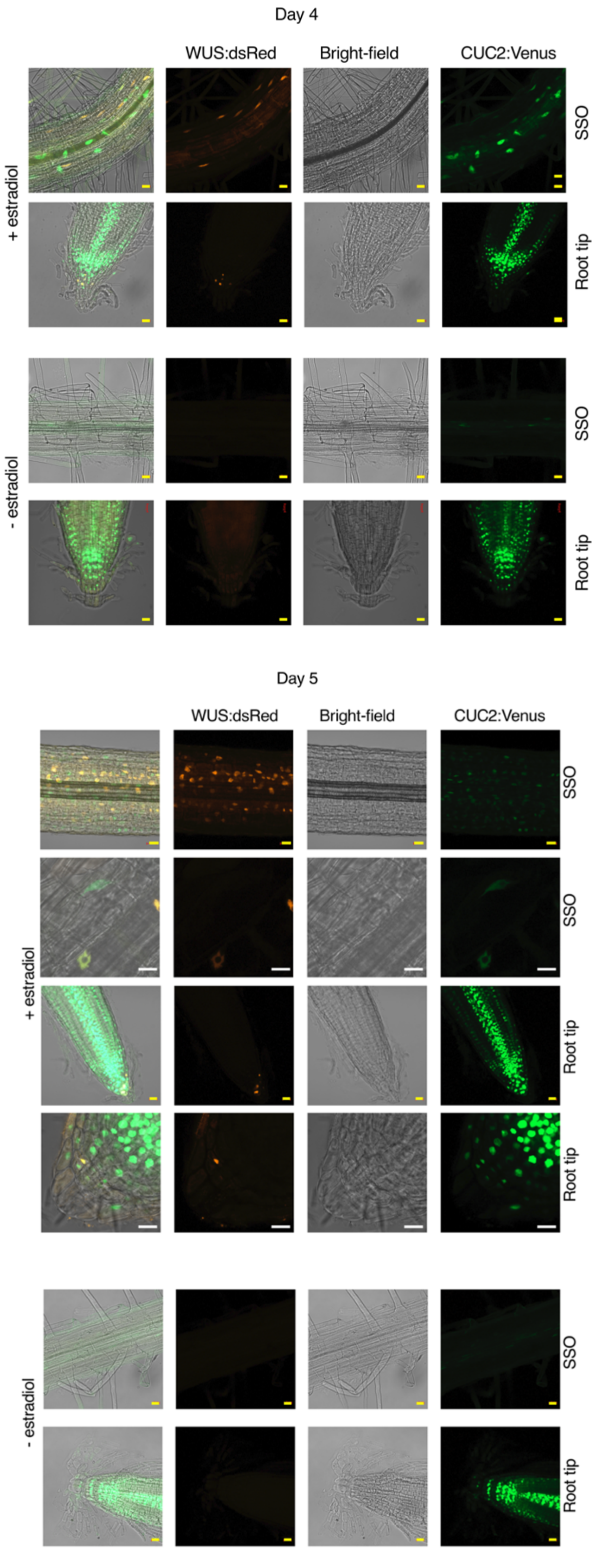

Figure S11. Expression of *CUC2* and *WUS* genes during SSO induction, Related to Figure 5. The *CUC2* and *WUS* gene expressions in the root of ATHB25/REM7 F1 seedlings harboring *pCUC2*:*VENUS* (Green) and *pWUS*:*dsRED* (Orange) during the SSO induction. The overlap of *CUC2* and *WUS* expression was represented as yellow. Magnified images of the *CUC2* and *WUS* expressions in SSO and root tip in day 4 and day 5 after the induction with or without the estradiol. Some figures are selected and used as duplicated in Figure 5. Scale bars: 20 µm.Scale bars: 20 µm.

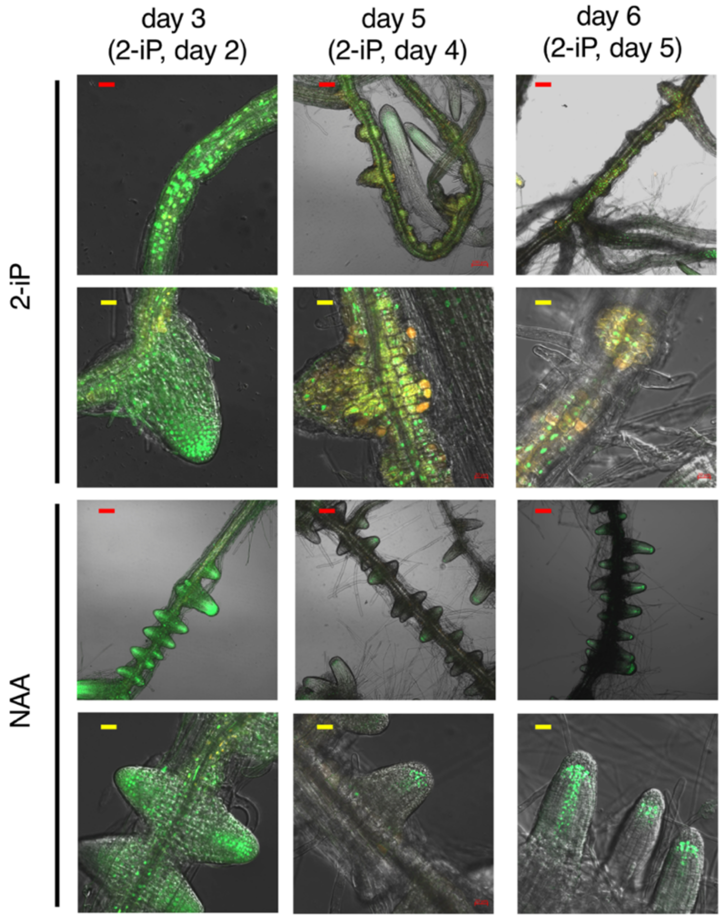

Figure S12. Expression of *CUC2* and *WUS* genes in shoot-to root conversion with phytohormones, Related to Figure 5. The *CUC2* and *WUS* gene expressions in the root of the seeding harboring *pCUC2*:*VENUS* (Green) and *pWUS*:*dsRED* (Orange) were observed in the pre-existing root-to-shoot conversion with phytohormones (Rosspopoff et al., 2017). The overlap of *CUC2* and *WUS* expression was represented as yellow. Cytokinin 2-iP treatment was performed after 42 h-NAA treatment. Scale bars: 20 µm (yellow), 100 µm (red).

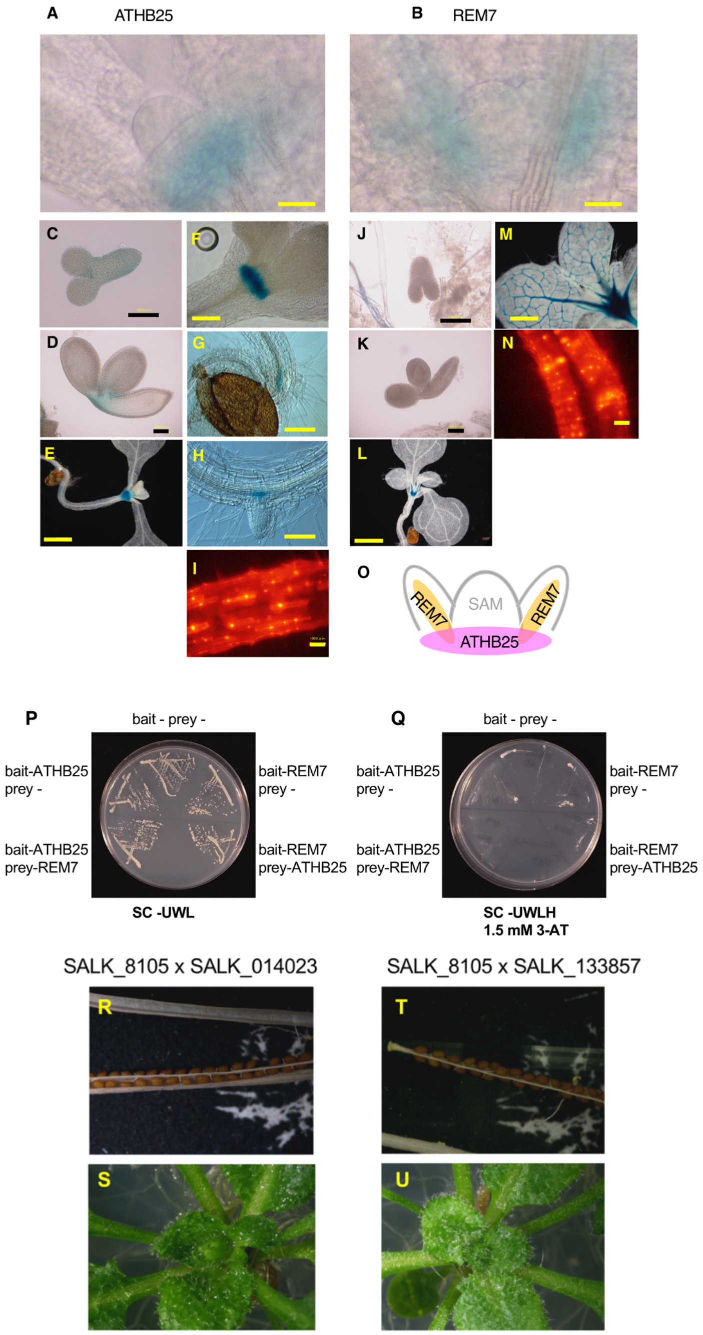

Figure S13. Expression, protein interaction, and loss-of-function phenotypes of ATHB25 and REM7, Related to Figure 6.

(A, C-I) ATHB25 gene expression and intracellular localization. (B, J-N) REM7 gene expression and intracellular localization. The patterns of promoter:GUS expression around the SAM of 7 day old seedling (A, B), in the embryo (C, D, I, J), around the SAM of 7 day old seedling (E, L), and of 2 week old seedling (F, M), in the border between hypocotyl and root (G), and in the boundary region of a lateral root of a mature plant (H). (I, N) Nuclear localization of the overexpressed mRFP (red fluorescent protein) fusion proteins of ATHB25 (I) and REM7 (N) in the root. Scale bars are 25 µm (A, B), 100 µm (C, D, I, J, K, M); 200 µm (F, G, H, M); and 1 mm (E, L). (P, Q) Protein interactions between ATHB25 and REM7 in yeast. We did not observe any obvious yeast growth on the SC-UWLH plates (Q), which strongly suggests there was no physical interaction between ATHB25 and REM7. (R, S) SALK_008105C x SALK_014023C homologous F2 plants (T, U) SALK_008105C x SALK_133857C homologous F2 plants. (R, T) Seeds in the siliques. (S, U) SAMs.

Table S1. SAM-specific Transcription factors, Related to Figure 1, S1, and S2. A total of 21 candidate genes encoding transcription factors (TFs) was selected in this study. Seven of 21 the genes were selected in the Confeito analysis with Network Factor > 0.75, and others were chosen in the ATTED II analysis. RIKEN full-length cDNAs were available for the nine candidates when we performed the cloning. Three series, Set A, B and C, of DNA fragments with estradiol inducible promoters for each cDNA, were cloned in the PRESSO method. Each cDNA was labeled as A to I in Figure S1B and S2C.

AGI code	Gene name	Network Factor (NF)	RAFL cDNA	Set	Supplementary Figs
At1g14440	ATHB31	0.791	RAFL09-75-E05	Set A	B
At1g75240	ATHB33	0.791	RAFL16-33-K14	Set A	E
At2g45190	FIL	0.778	RAFL16-22-D15	Set A	D
At3g18960	REM7	0.791	RAFL21-04-N20	Set C	I
At3g50890	ATHB28	-	RAFL22-13-N07	Set A	C
At3g60390	HAT3	-	RAFL09-45-G03	Set A	A
At3g61310	AHL11	0.791	RAFL07-16-A05	Set B	F
At4g31805	POLAR	-	RAFL14-02-E14	Set B	G
At5g65410	ATHB25	0.791	RAFL09-18-O11	Set C	H
At3g61830	ARF18	0.776			
At2g02540	AHB21	-			
At1g73360	EDT1/HDG11	-			
At4g21750	ATML1	-			
At1g17920	HDG12	-			
At2g37630	AS1	-			
At4g00180	YAB3	-			
At3g14980	IDM1/ROS4	-			
At5g46880	HDG5/ATHB7	-			
At4g04890	PDF2	-			
At3g61250	MYB17/LMI2	-			
At5g03790	ATHB51/LMI1	-			

Table S2. Primers used for cloning in this study, Related to Figure 1, S1, and S2.

Primer name	Primer sequence	target genes
hsp-ter_FW_SfiI	5'-GAGTGGACGATTGGCAGAAGGCCATAAAGGCCATATGAAGATGAAGATGAAATATTTGGTG-3'	hsp terminator
hsp-ter_RV_XhoI	5'-GAGTGGACGATTGGCAGAAGCTCGAGCTTATCTTTAATCATATTCCATAGTCCAT-3'	hsp terminator
LexA_ FW_XhoI	5'-GAGTGGACGATTGGCAGAAGCTCGAGCCCCTCGACAGCTTGCAT-3'	LexA operator
LexA_RV_SfiI	5'-GAGTGGACGATTGGCAGAAGGCCAATTAGGCCAGGATCCGACTAGCTTCA-3'	LexA operator
CSPS_FW_SfiI	5'-GAGTGGACGATTGGCAGAAGGCCAAATAGGCCTGGCAAACAGCTATTATGGGTATTATGG-3'	SfiI and I-SceI site
CSPS_RV_I-SceI	5'-GAGTGGACGATTGGCAGAAGTAGGGATAACAGGGTAATTCTTCTTCG-3'	SfiI and I-SceI site
pUC_FW	5'-GAGTGGACGATTGGCAGAAGCAGCTGGCACGACAGGTTT-3'	pHSG299CSPS vector backbone
pUC_ RV_ SfiI_I-SceI	5'-AAGGAAAAAAGGCCATTAAGGCCTAGGGATAACAGGGTAATTCTTCTTCG-3'	pHSG299CSPS vector backbone
35S_ FV_ SfiI	5'-GAGTGGACGATTGGCAGAAGGCCTGCATGGCCGTTTTCCCAGTCACGACGTT-3'	35S promoter:Ω
35S_RV_SfiI	5'-GAGTGGACGATTGGCAGAAGGCCAATTAGGCCAAACTTGTTGATAACTCTAGAAATTG-3'	35S promoter:Ω
XVE_ FW_ SfiI	5'-GAGTGGACGATTGGCAGAAGGCCAAATCGGCCATGAAAGCGTTAACGGCCAG-3'	XVE
XVE_RV_SfiI	5'-GAGTGGACGATTGGCAGAAGGCCCTTATGGCCAAGCTTGTTTGGGATGTT-3'	XVE
hsp-ter_FW2_SfiI	5'-TCCCTAGGCCTTAAAGGCCATATGAAGATGAAGATGAAATATTTGGTG-3'	the vector backbone
I-SceI RV.	5'-GAGTGGACGATTGGCAGAAGTAGGGATAACAGGGTAATTCTTCTTCG-3'	the vector backbone
OP1_FW_HindIII	5'-CCCCAAGCTTAGCTTGGGCTGCAGGTCGAG-3'	LexA operator and minimum promoter
OP1_RV_ HindIII	5'-CCCCAAGCTTGACTAGCTTCAGCGTGTCCT-3'	LexA operator and minimum promoter
hsp-ter_FW_SacI	5'-CCCCGAGCTCATATGAAGATGAAGATGAAA-3'	hsp terminator
hsp-ter_RV_SacI	5'-CCCCGAGCTCCTTATCTTTAATCATATTCC-3'	hsp terminator
35S-XVE_FW_AscI	5'-AAAAGGCGCGCCCAACATGGTGGAGCACGACA-3'	35S:Ω:XVE:E9 terminator
35S-XVE_ RV_AscI	5'-AAAAGGCGCGCCGTTTGGGATGTTTTACTCCT-3'	35S:Ω:XVE:E9 terminator
ATHB25_CDS_FW	5'-AAAAAGCAGGCTTCATGGAGTTTGAAGACAACAACAACA-3'	ATHB25
ATHB25_CDS_RV	5'-AGAAAGCTGGGTGTCATGGTTGGTCTTGTTCATGATG-3'	ATHB25
REM7_CDS_FW	5'-AAAAAGCAGGCTTCATGGTTACAACCCAAAACACGAAG-3'	REM7
REM7_CDS_RV	5'-AGAAAGCTGGGTGTTATCCCCTGAAGACTCTCTTGT-3'	REM7
attB1	5'-GGGGACAAGTTTGTACAAAAAAGCAGGCTTC-3'	extend the overhang sequences for Gateway Cloning.
attB2	5'-GGGGACCACTTTGTACAAGAAAGCTGGGTG-3'	extend the overhang sequences for Gateway Cloning.
ATHB25_promoter_FW	5'-AAAAAAGCAGGCTCACTTGCAATTTTATAAAATTGTGAGA-3'	ATHB25 promoter
ATHB25_promoter_RV	5'-ACAAGAAAGCTGGGTCCATTCAAGAAGTCGAGAAATG-3'	ATHB25 promoter
REM7_promoter_FW	5'-AAAAAAGCAGGCTCACCATACAATCTTACTCTCTAAATTCC-3'	REM7 promoter
REM7_promoter_RV	5'-ACAAGAAAGCTGGGTCCATTTTTGTTTGCCTTGTTCAG-3'	REM7 promoter

Table S3. Primers used for RT-qPCR in this study, Related to Figure 1 and S2.

Primer name	Primer sequence	target genes
ATHB25_RT-FW	5'-CAACAACAACAACAACGACGAA-3’	ATHB25
ATHB25_RT-RV	5'-AGAACACGAGAGAGAGGAGGAGAG-3’	ATHB25
REM7_RT-FW	5'-GCTTGCGAGACCAAATATCCA-3’	REM7
REM7_RT-RV	5'-CCCTGAAGACTCTCTTGTCTTCTTC-3’	REM7
UBQ10_FW	5'-GAAGTTCAATGTTTCGTTTCATGT-3’	UBQ10
UBQ10_RV	5'-GGATTATACAAGGCCCCAAAA-3’	UBQ10

Table S4. Gene Enrichment Analysis for the target genes regulated by ATHB25 and REM7, Related to Figure 4. GO enrichment analysis was performed with >10-fold regulated genes using the AmiGo2 browser (http://amigo.geneontology.org/amigo) on the Gene Ontology Consortium website (The Gene Ontology Consortium, 2015). GO biological processes enriched in the ATHB25-ind, REM7-ind, and ATHB25/REM7-ind plants are listed with a threshold of 10-fold changes in the Table.

**a**, The biological processes up-regulated in the both TF-ind plants		
GO biological process complete	fold Enrichment	P-value
regulation of vitamin metabolic process (GO:0030656)	> 100	9.55E-03
petal epidermis patterning (GO:0080172)	> 100	9.55E-03
regulation of L-ascorbic acid biosynthetic process (GO:2000082)	> 100	9.55E-03
putrescine metabolic process (GO:0009445)	52.12	1.90E-02
malate transmembrane transport (GO:0071423)	52.12	1.90E-02
cutin transport (GO:0080051)	52.12	1.90E-02
ornithine metabolic process (GO:0006591)	34.74	1.59E-03
glycyl-tRNA aminoacylation (GO:0006426)	34.74	2.84E-02
cellular response to sucrose starvation (GO:0043617)	34.74	2.84E-02
glucosylceramide catabolic process (GO:0006680)	26.06	3.77E-02
glucosylceramide metabolic process (GO:0006678)	26.06	3.77E-02
negative regulation of apoptotic process (GO:0043066)	26.06	3.77E-02
threonyl-tRNA aminoacylation (GO:0006435)	26.06	3.77E-02
UDP-N-acetylglucosamine biosynthetic process (GO:0006048)	26.06	3.77E-02
amino sugar biosynthetic process (GO:0046349)	26.06	3.77E-02
regulation of apoptotic process (GO:0042981)	26.06	3.77E-02
urea cycle (GO:0000050)	20.85	4.68E-02
UDP-N-acetylglucosamine metabolic process (GO:0006047)	20.85	4.68E-02
cellular response to hypoxia (GO:0071456)	19.85	5.72E-05
cellular response to oxygen levels (GO:0071453)	18.13	8.11E-05
cellular response to decreased oxygen levels (GO:0036294)	18.13	8.11E-05
**b**, The biological processes down-regulated in the both TF-ind plants		
GO biological process complete	fold Enrichment	P-value
cellular response to fatty acid (GO:0071398)	> 100	6.83E-03
response to fatty acid (GO:0070542)	> 100	6.83E-03
protein ADP-ribosylation (GO:0006471)	> 100	9.10E-03
developmental programmed cell death (GO:0010623)	72.91	1.36E-02
DNA ligation involved in DNA repair (GO:0051103)	72.91	1.36E-02
lagging strand elongation (GO:0006273)	62.49	1.59E-02
DNA ligation (GO:0006266)	62.49	1.59E-02
DNA strand elongation involved in DNA replication (GO:0006271)	48.6	2.04E-02
DNA strand elongation (GO:0022616)	48.6	2.04E-02
anther wall tapetum development (GO:0048658)	48.6	2.04E-02
**c**, The biological processes up-regulated in the ATHB25-ind plants		
GO biological process complete	fold Enrichment	P-value
cellular response to hypoxia (GO:0071456)	54.54	1.54E-11
cellular response to oxygen levels (GO:0071453)	49.79	3.78E-11
cellular response to decreased oxygen levels (GO:0036294)	49.79	3.78E-11
response to hypoxia (GO:0001666)	23.86	4.90E-08
response to oxygen levels (GO:0070482)	21.21	1.52E-07
response to decreased oxygen levels (GO:0036293)	21.21	1.52E-07
hydrogen peroxide catabolic process (GO:0042744)	11.26	3.55E-02
hydrogen peroxide metabolic process (GO:0042743)	10.91	4.25E-02
**d**, The biological processes down-regulated in the ATHB25-ind plants		
GO biological process complete	fold Enrichment	P-value
nuclear transport (GO:0051169)	29.26	3.36E-02
nucleocytoplasmic transport (GO:0006913)	29.26	3.36E-02

**e**, The biological processes up-regulated in the REM7-ind plants		
GO biological process complete	fold Enrichment	P-value
cellular response to hypoxia (GO:0071456)	> 100	2.71E-06
cellular response to oxygen levels (GO:0071453)	98.32	4.26E-06
cellular response to decreased oxygen levels (GO:0036294)	98.32	4.26E-06
response to hypoxia (GO:0001666)	47.11	1.62E-04
response to oxygen levels (GO:0070482)	41.88	2.89E-04
response to decreased oxygen levels (GO:0036293)	41.88	2.89E-04

**f**, The biological processes down-regulated in the REM7-ind plants		
GO biological process complete	fold Enrichment	P-value
negative regulation of catalytic activity (GO:0043086)	31.77	3.10E-02
negative regulation of molecular function (GO:0044092)	28.88	3.41E-02
transmembrane receptor protein tyrosine kinase signaling pathway (GO:0007169)	23.66	4.15E-02
enzyme linked receptor protein signaling pathway (GO:0007167)	23.41	4.19E-02

KEY RESOURCE TABLE

REAGENT or RESOURCE	SOURCE	IDENTIFIER
Bacterial Strain		
Agrobacterium tumefaciens EHA105	N/A	N/A
One Shot™ ccdB Survival™ 2 T1R Competent Cells	Thermo Fisher Scientific	https://www.thermofisher.com
E. coli DH5α Competent Cells	TaKaRa Bio	http://www.takara-bio.co.jp
Saccharomyces cerevisiae L40	N/A	N/A
Chemicals		
Murashige and Skoog Basal Salt Mix	Sigma-Aldrich	Cat# M5524
Murashige and Skoog Vitamin 1000 x liquid	Sigma-Aldrich	Cat# M3900
Sucrose	Nacalai	Cat# 30403
Kanamycin	Wako Chem.	Cat# 133-93-6
Hygromycin B	Nacalai	Cat# 31282-04-9
Rifampicin	Sigma-Aldrich	Cat# 13292-46-1
Ampicilin	Wako Chem.	Cat# 69-52-3
Spectinomycin Dihydrochloride Pentahydrate	Wako Chem.	Cat# 22189-32-8
Gentamicin Sulfate	Wako Chem.	Cat# 1405-41-0
Phytoagar	DUCHEFA	Cat# P1003
Phytagel	Wako Chem.	Cat# 71010-52-1
17-ß-estradiol	Sigma-Aldrich	Cat# 57-63-6
6-benzyladenine (BA)	Sigma-Aldrich	Cat# 1214-39-7
5-bromo-4-chloro-3-indolyl-β-D-glucuronide cyclohexylammonium salt (X-Gluc)	Wako Chem.	Cat# 114162-64-0
Tris (hydroxymethyl) aminomethane	Nacalai	Cat# 35401-25
EDTA	Nacalai	Cat# 15108-05
Lithium acetate	Wako Chem.	Cat# 546-89-4
Polyethylene Glycol 4000	Wako Chem.	Cat# 25322-68-3
Dimethyl sulfoxide	Wako Chem.	Cat# 67-68-5
Potassium Hexacyanoferrate (III)	Wako Chem.	Cat# 13746-66-2
Potassium Hexacyanoferrate (II) Trihydrate	Wako Chem.	Cat# 14459-95-1
Potassium Dihydrogenphospate	Nacalai	Cat# 7778-77-0
di-Potassium Hydrogenphosphate	Nacalai	Cat# 28727-95
Bacto-yeast extract	BD Biosciences	Cat# 212750
Bacto-peptone	BD Biosciences	Cat# 211677
Glucose	Nacalai	Cat# 16805-35
Yeast Nitrogen Base without Amino Acids	Difco	Cat# DF0919-15-3
L-Arginine	Wako Chem.	Cat# 74-79-3
L-isoleucine	Wako Chem.	Cat# 73-32-5
L-Lysine	Wako Chem.	Cat# 56-87-1
L-Methionine	Wako Chem.	Cat# 63-68-3
L-Phenylalanine	Wako Chem.	Cat# 63-91-2
L-Tyrosine	Wako Chem.	Cat# 60-18-4
Adenine Sulfate	Wako Chem.	Cat# 321-30-2
Uracil	Wako Chem.	Cat# 66-22-8
L-Tryptophan	Wako Chem.	Cat# 73-22-3
L-Leucine	Wako Chem.	Cat# 61-90-5
L-Histidine	Wako Chem.	Cat# 71-00-1
3-AT	Wako Chem.	Cat# 61-82-5
Restriction Enzymes	TaKaRa Bio	http://www.takara-bio.co.jp
Restriction Enzymes	TOYOBO	http://www.toyobo-global.com/seihin/xr/lifescience/
PrimeSTAR HS DNA Polymerase	TaKaRa Bio	http://www.takara-bio.co.jp
PrimeSTAR GXL DNA polymerase	TaKaRa Bio	http://www.takara-bio.co.jp
Critical Commercial Assays and Kit		
Gateway BP Clonase II Enzyme mix	Thermo Fisher Scientific	https://www.thermofisher.com
Gateway LR Clonase II Enzyme mix	Thermo Fisher Scientific	https://www.thermofisher.com
MultSite Gateway Cloning Kit	Thermo Fisher Scientific	https://www.thermofisher.com
QIAquick Gel Extraction Kit	QIAGEN	http://www.qiagen.com
QIAprep Spin Miniprep Kit	QIAGEN	http://www.qiagen.com
DNA Ligation Kit ＜Mighty Mix＞	TaKaRa Bio	http://www.takara-bio.co.jp
TaKaRa DNA Ligation Kit LONG	TaKaRa Bio	http://www.takara-bio.co.jp
RNeasy Plant Mini Kit	QIAGEN	http://www.qiagen.com
Low Input Quick Amp Labeling Kit (one color, Cyanine3-CTP)	Agilent Technologies	www.agilent.com
Agilent Arabidopsis Oligo DNA Microarray Ver. 4	Agilent Technologies	www.agilent.com
Gene Expression Hybridization Kit	Agilent Technologies	www.agilent.com
RNA Spike In Kit	Agilent Technologies	www.agilent.com
SuperScript III First-Strand Synthesis System	Thermo Fisher Scientific	https://www.thermofisher.com
DyNAmo HS SYBR Green qPCR kit	Thermo Fisher Scientific	https://www.thermofisher.com

Experimental Models: Organisms/Strains		
Arabidopsis thaliana ecotype Columbia	Rhee et al., 2003	https://abrc.osu.edu
Arabidopsis thaliana 9 genes	This study	N/A
Arabidopsis thaliana 4 genes (set B&C)	This study	N/A
Arabidopsis thaliana 2 genes (set B)	This study	N/A
Arabidopsis thaliana 2 genes [set C (ATHB25/REM7-ind)]	This study	N/A
Arabidopsis thaliana ATHB25-ind (pGWB501_TOPX_ATHB25)	This study	N/A
Arabidopsis thaliana REM7-ind (pGWB501_TOPX_REM7)	This study	N/A
Arabidopsis thaliana ATHB25-ox (pGWB502Ω_ATHB25)	This study	N/A
Arabidopsis thaliana REM7-ox (pGWB502Ω_REM7)	This study	N/A
Arabidopsis thaliana ATHB25Pro:GUS (pGWB533_ATHB25pro)	This study	N/A
Arabidopsis thaliana REM7Pro:GUS (pGWB533_REM7pro)	This study	N/A
Arabidopsis thaliana 35S:mRFP-ATHB25 (pGWB555_ATHB25)	This study	N/A
Arabidopsis thaliana pCUC2:3xVenus-N7 pWUS:dsRed-N7	Heisler et. al., 2005, Reddy et al., 2005	https://abrc.osu.edu
Arabidopsis thaliana pWOX4:GUS	Hirakawa et al., 2010	https://abrc.osu.edu
Arabidopsis thaliana 35S:mRFP-REM7 (pGWB555_REM7)	This study	N/A
Arabidopsis thaliana T-DNA tagged line SALK_133857C	Alonso et al., 2003	http://signal.salk.edu
Arabidopsis thaliana T-DNA tagged line SALK_014023C	Alonso et al., 2003	http://signal.salk.edu
Arabidopsis thaliana T-DNA tagged line SALK_008105C	Alonso et al., 2003	http://signal.salk.edu
Origonucleotides		
Primers are listed in Table S2 and S3	This study	N/A
Recombinant DNA		
RIKEN Arabidopsis full-length cDNA clones are listed in Table S1	Seki et al., 2004	http://epd.brc.riken.jp/en/pdna/rafl_clones
pER8	Zuo et al., 2000	N/A
pGWB501	Nakagawa et al., 2009	N/A
pGWB502Ω	Nakagawa et al., 2009	N/A
pGWB533	Nakagawa et al., 2009	N/A
pGWB555	Nakagawa et al., 2009	N/A
pDEST-BTM116	Mitsuda et al., 2010	N/A
pDEST-GAD424	Mitsuda et al., 2010	N/A
pHSG299CSPS	Fujisawa et al., 2009	N/A
pDONR Zeo Thsp R3 NPTII R4 L2 ccdB L1 CSPS	This study	N/A
pGWB501_TOPX	This study	N/A
pENTR-Gus	Thermo Fisher Scientific	https://www.thermofisher.com
pDONR 221	Thermo Fisher Scientific	https://www.thermofisher.com
pGWB501_TOPX-GUS	This study	N/A
pGWB501_9_genes (set #21)	This study	N/A
pGWB501_4_genes (105B_105I_XVE) set B&C	This study	N/A
pGWB501_2_genes (105I_XVE) setB	This study	N/A
pGWB501_2_genes (105B_XVE) setC	This study	N/A
pGWB501_TOPX_ATHB25	This study	N/A
pGWB501_TOPX_REM7	This study	N/A
pGWB502Ω_ATHB25	This study	N/A
pGWB502Ω_REM7	This study	N/A
pGWB555_ATHB25	This study	N/A
pGWB555_REM7	This study	N/A
pGWB533_ATHB25pro	This study	N/A
pGWB533_REM7pro	This study	N/A
pDEST_BTM116_REM7	This study	N/A
pDEST_BTM116_ATHB25	This study	N/A
pDEST_GAD424_REM7	This study	N/A
pDEST_GAD424_ATHB25	This study	N/A

TRANSPARENT METHODS

EXPERIMENTAL MODEL AND SUBJECT DETAILS

We used *Arabidopsis thaliana* ecotype Columbia, available from the Arabidopsis Biological Resource Centre (ABRC, Ohio State University, https://abrc.osu.edu) (Rhee et al., 2003). The constructs in binary vectors in the 'Plasmid Construction' section were introduced into Arabidopsis using *Agrobacterium tumefaciens* (updated scientific name, *Rhizobium radiobacter*) EHA105 (Clough and Bent, 1998) and T1 transformants were selected on 1/2 Murashige-Skoog (MS)-1% phytoagar medium containing 1% sucrose (pH 5.7) with the appropriate antibiotic (25 μg/L kanamycin or 20 μg/L hygromycin). ATHB25/REM7-ox plants and ATHB25/REM7-ind plants harboring *CUC2*, *WUS*, or *WOX4*:reporter genes (Heisler et al., 2005; Hirakawa et al., 2010; Reddy and Meyerowitz, 2005) were generated by artificial pollination.

Growth conditions

Seeds were surface sterilized with a 70% ethanol rinse, immediately followed by a rinse with 33% bleach for 5 min and then washed twice with sterile water. The seeds were sown aseptically on antibiotic-containing 1/2 MS-1% agar plates, followed by imbibition at 4°C for 3–4 days in the dark. Seedlings were grown for seven days at 22°C under constant light conditions or 16 h light: 8 h dark cycles with 40 μmol m^{-2} sec^{-1} cool white fluorescent light. The antibiotic-resistant seedlings were transferred to 1/2 MS-1.2% phytagel or 1% phytoagar plates without antibiotics, with 1–10 μM 17-ß-estradiol used for the chemical induction and 1 μM 6-benzyladenine (BA) for the cytokinin treatment. For observation of the SSO formation, the plants were grown on the surface of the 1/2 MS plates in a vertical placement. To induce lateral root formation, plants were transferred and grown for 42 hours on 1/2 MS-1% agar plates containing the auxin 1-naphthaleleacetic acid (10 μM NAA). To induce the subsequent root-to-shoot conversion, primary root segments were transferred on the 1/2 MS-1% agar plates containing the cytokinin 2-isopentenyladenine (8.16 μM 2-iP) (Rosspopoff et al., 2017).

Bioinformatic analysis for candidate TF genes

To classify TFs that cooperate and co-express in plants, we analyzed approximately 2000 genes encoding Arabidopsis TFs using the Confeito algorithm (Ogata et al., 2010) (Figure S1A-C). Confeito analysis was performed on our website (Cop: Co-expressed biological processes, http://webs2.kazusa.or.jp/kagiana/cop0911/). The Confeito algorisms provided 21 sets of TF genes, which are clustered based on their co-expression patterns (Network Factor (NF) >0.75). In this study, we focused on a set consisting of seven TFs, ATHB31, ATHB33, ABNORMAL FLORAL ORGANS (AFO), REM7, AT-HOOK MOTIF NUCLEAR LOCALIZATION PROTEIN 11 (AHL11), AUXIN RESPONSE FACTOR 18 (ARF18) and ATHB25, which are co-expressed around the shoot apical meristems (SAMs). The AGI codes of the genes belonging to this set are shown with NF in Table S1. The details of these genes are available in the public database, The Arabidopsis Information Resource (TAIR, http://www.arabidopsis.org) (Huala et al., 2001). In the Confeito analysis, some genes were not clustered in any sets when their expression data were not available on the database. As the Confeito algorism provided the compact sets of transcription factors that were clustered based on the co-expression data, if the spatiotemporal expressions of genes were more specifically limited, the known SAM-specific TF genes such as *WUS* were classified in other groups. For example, WUS is grouped in another set consisting of SHINE 1 (SHN1, At1g15360), BIG PETAL (BPE, At1g59640), NAC DOMAIN CONTAINING PROTEIN 25 (NAC25, At1g61110), AGAMOUS (AG, At4g18960), PISTILLATA (PI, At5g20240) and NAC DOMAIN CONTAINING PROTEIN 100 (NAC100, At5g61430). To identify more genes that co-related to the seven TFs in the set we focused on, we screened for TF genes co-expressing with the seven candidates by ATTED II analysis (http://atted.jp) (Obayashi et al., 2014). We added 14 TF genes with the mutual rank of <46, which was that of At3g61830, as the threshold in this cluster (Table S1). A total of 21 candidate genes were selected (Figure S1A).

Plasmid Construction

We obtained nine full-length cDNAs from these candidates from the RIKEN BioResource Center (www.brc.riken.jp) (Seki et al., 2004) (Table S1: Figure S1A-C). The other 12 cDNAs were not available at the time when this study was performed. To co-induce the nine SAM-specific TFs *in planta*, we sequentially and alternately ligated

these RIKEN full-length cDNAs(Seki et al., 2004) to the terminator-operator cassettes consisting of the Arabidopsis *heat-shock protein 18.2* (*hsp*) gene terminator, the 8x *Lex*A operator (XVE)(Zuo et al., 2000) (Moore et al., 2006) and the cauliflower mosaic virus 35S (CaMV 35S) minimal promoter using the PRESSO (precise sequential DNA ligation on a solid substrate) method (Takita et al., 2013) and MultiRound Gateway technology (Thermo Fisher Scientific, Wilmington, USA, https://www.thermofisher.com/) (Chen et al., 2006). We successfully cloned the nine cDNAs under the control of the estradiol-inducible XVE expression cassette in a vector as shown below (Figure S1B). Plasmids for ATHB25-ind, REM7-ind, ATHB25/REM7-ind, ATHB25-ox, REM7-ox, ATHB25-promoter:GUS, REM7-promoter:GUS, 35S:Ω:mRFP:ATHB25 and 35S:Ω:mRFP:REM7 plants were obtained by the Gateway Cloning reaction (Thermo Fisher Scientific, Wilmington, USA, https://www.thermofisher.com/). Unless otherwise noted, polymerase chain reactions (PCR) for subcloning were performed using PrimeSTAR HS DNA Polymerase (TaKaRa Bio Inc., Shiga, Japan, http://www.takara-bio.co.jp/). The details of plasmid cloning are described in below.

First, we prepared an Arabidopsis heat shock protein 18.2 (*hsp*) terminator/*Lex*A operator/-46 to +12 of the cauliflower mosaic virus (CaMV) 35S promoter fragment (TOP)-cassette in the pHSG299CSPS vector (Fujisawa et al., 2009). This cassette terminates transcription of the upstream gene and chemically induces the downstream genes. The DNA fragments of (i) *hsp* terminator, (ii) *Lex*A operator and – 46 to +12 of the CaMV 35S promoter, (iii) *Sfi*I site and (iv) the whole of the pHSG299CSPS vector backbone without a multicloning site were amplified from pER8 and pHSG299CSPS. PCR amplification was performed with the following primer sets (Table S2): hsp-ter_FW_SfiI and hsp-ter_RV_XhoI for *hsp* terminator; LexA_ FW_XhoI and LexA_RV_SfiI for *Lex*A operator; CSPS_FW_SfiI and CSPS_RV_I-SceI for *Sfi*I and I-*Sce*I site; pUC_FW and pUC_ RV_ SfiI_I-SceI for pHSG299CSPS vector backbone. Each of the PCR products was digested with suitable restriction enzymes and sequentially ligated using the PRESSO method (Takita et al., 2013), in the following order:*hsp* terminator, *Lex*A operator and -46 to +12 of CaMV 35S promoter and pHSG299CSPS vector backbone without a multi-cloning site. The resultant plasmid

provided the TOP-cassette between two *Sfi*I sites (GGCCTTAAAGGCC and GGCCTAATAGGCC).

We also cloned a DNA fragment encoding a chimeric transcription activator (XVE) that consists of the DNA-binding domain of the bacterial repressor *Lex*A (X), the acidic transactivation domain of VP16 (V) and the regulatory region of the human estrogen receptor (E) (XVE) under the 2 x CaMV 35S promoter:Ω transcriptional enhancer (35S promoter:Ω). The XVE and 35S promoter:Ω DNA fragments were amplified from pER8 (Zuo et al., 2000) and pGWB502Ω (Nakagawa et al., 2009) respectively, and cloned into the pDONR-based vector, pDONR Zeo Thsp R3 NPTII R4 L2 ccdB L1 CSPS (Accession Number: LC217877) by using the PRESSO method, in the following order: I-*Ceu*I, I-*Sce*I, PI-*Psp*I, PI-*Sce*I, *Sfi*I restriction enzyme recognition sites, 35S promoter:Ω:XVE in the vector. The following primer sets were used for PCR amplification (Table S2): The CSPS_FW_SfiI and CSPS_RV_I-SceI for the I-*Ceu*I, I-*Sce*I, PI-*Psp*I, PI-*Sce*I, *Sfi*I restriction enzyme recognition sites, described in the previous section; 35S_ FV_ SfiI and 35S_RV_SfiI for 35S promoter:Ω; XVE_ FW_ SfiI and XVE_RV_SfiI for XVE; hsp-ter_FW2_SfiI and I-SceI RV for the vector backbone. The resultant plasmid provided the 35S promoter:Ω:XVE cassette.

To achieve the chemical induction of the SAM-specific transcription factors, we prepared nine full-length cDNA fragments encoding the SAM-specific TFs and TOP-cassette DNA fragments. The list of RAFL cDNA clones used was shown in Table S1. Each RAFL cDNA that was inserted between the two *Sfi*I sites (GGCCAAATCGGCC and GGCCATAAGGGCC) in the modified BluescriptII vector (Seki et al., 2004) was digested by *Sfi*I, electrophoresed to separate full-length cDNA fragments and purified using the QIAquick Gel Extraction Kit (QIAGEN GmbH, Hilden, Germany, http://www.qiagen.com). The TOP-cassette was also digested by *Sfi*I and purified. First, a TOP cassette was ligated to the spacer DNA on the streptavidin-beads in the PRESSO method (Takita et al., 2013), and then a full-length cDNA fragment was ligated to the TOP cassette on the beads. Multiple cDNA fragments and TOP cassettes were reciprocally ligated to the DNA fragment on the beads in a repetitive manner in the following order: [TOP, At3g60390, TOP, At1g14440, TOP, At3g50890, TOP,

At2g45190, TOP and At1g75240 (**set A**)], [TOP, At3g61310, TOP and At4g31805 (**set B**)], and [TOP, ATHB25, TOP, and REM7 (**set C**)] (Table S1). In the last step of the PRESSO, we ligated the pDONR-based vector DNA fragments, pDONR Zeo Thsp R3 NPTII R4 L2 ccdB L1 CSPS, containing an I-*Sce*I restriction-enzyme-recognition site that was amplified with a PrimeSTAR GXL DNA polymerase (TaKaRa Bio Inc.). The bead-bound DNA fragments were digested with the restriction enzyme I-*Sce*I. The recovered DNAs were purified and self-ligated via the I-*Sce*I site. Finally we generated three sets of multiple cDNAs in the entry vector [**set A**: At3g60390, At1g14440, At3g50890, At2g45190 and At1g75240, **set B**: At3g61310 and At4g31805, and **set C**: ATHB25 (At5g65410) and REM7 (At3g18960)] under the control of the *Lex*A operator in the pDONR-based vector for Gateway Cloning using PRESSO (Chen et al., 2006; Takita et al., 2013) (Figure S2C).

We amplified these ligated cassettes using the TaKaRa DNA Ligation Kit LONG (TaKaRa Bio Inc.) and then combined these inserts with the MultiRound Gateway Cloning kit (Thermo Fisher Scientific)(Chen et al., 2006). We also cloned individual genes, 'set B', 'set C' and the combined fragment 'set B' and 'set C' into the chemically inducible vector pGWB501_TOPX, which is constructed in the following section (Figure S2C).

To prepare an estradiol-inducible Gateway vector (pGWB501_TOPX), we amplified the 'LexA operator, -46 to +12 of 35S promoter', 'Hsp18.2 terminator' and '35S promoter-omega translational enhancer: XVE fusion protein, rat glucocorticoid receptor 3' UTR pea rbcS E9 terminator' DNA fragments from the plasmid DNAs described in the previous section, and cloned these fragments into the *Hind*III, *Sac*I and *Asc*I sites on pGWB501, respectively (Figure S2A). The following primer sets were used for the PCR amplification (Table S2): OP1_FW_HindIII and OP1_RV_ HindIII for *Lex*A operator and minimum promoter; hsp-ter_FW_SacI and hsp-ter_RV_SacI for hsp terminator; 35S-XVE_FW_AscI and 35S-XVE_ RV_AscI for 35S:Ω:XVE: E9 terminator. The resultant plasmid vector was termed 'pGWB501_TOPX' (Accession Number: LC217876). The pGWB501_TOPX vector contains an operator:minimum 35S promoter-gateway cassette-hsp terminator, 35S promoter-XVE enhancer gene-rbcS E9

terminator and a hygromycin-resistant gene for plants and a spectinomycin-resistant gene for bacteria. The XVE enhancer protein is activated by 17-ß-estradiol treatment, and then will transcribe the gene that is cloned in the Gateway cloning site.

To test whether the estradiol-inducible system facilitates expression of a foreign gene, we cloned the *ß-glucuronidase* (*GUS*) gene into the Gateway cloning site of pGWB501_TOPX and introduced the *GUS* gene with the *Lex*A operator into Arabidopsis (ecotype Columbia) plants via *Agrobacterium* using a floral dipping method (Clough and Bent, 1998). The *GUS* gene was provided by pENTR-Gus (Thermo Fisher Scientific). The T1 plants harboring pGWB501_TOPX-GUS were selected on 1/2 MS plates containing 25µg/L hygromycin. The T2 plants were grown for a week on hygromycin plates and then transferred to plates including 5 µM estradiol. The plants were grown for 10 days on the estradiol plates and then subjected to GUS staining (See the following section 'Glucuronidase analysis'). After treatment with estradiol, GUS staining was detected. We confirmed the chemical induction of gene expression in the pGWB501_TOPX-GUS plants (Figure S2B).

To evaluate the nine candidate genes, set B&C, set B, set C, ATHB25 and REM7 were cloned into the pGWB501_TOPX vector (Figure S2C). Clones for induction of 4 genes (set B&C), both genes simultaneously [set B and set C] and individually, ATHB25 (ATHB25-ind) and REM7 (REM7-ind), were produced. We did not analyze the set A construct. Individual sequences (CDS) encoding ATHB25 and REM7 and a combined set of both cDNAs (ATHB25/REM7) with chemically inducible promoters were cloned into the gateway vector pGWB501_TOPX (Figure S2C). Each CDS DNA fragment was amplified by PCR with the following primer sets overhanging *att*B1 or *att*B2 sequences (Table S2): ATHB25_CDS_FW and ATHB25_CDS_RV for ATHB25; REM7_CDS_FW and REM7_CDS_RV for REM7; attB1 and attB2 to extend the overhang sequences for Gateway Cloning. ATHB25/REM7 was constructed as "set C" in the entry vector by using the PRESSO method (Takita et al., 2013). The Gateway Cloning BP and LR reactions were performed following the manufacturer's recommendations (Thermo Fisher Scientific). We termed single ATHB25-, REM7- and "set C"-inducible clones as ATHB25-ind, REM7-ind and ATHB25/REM7-ind,

respectively. To confirm the ATHB25 and REM7 gene expression in the inducible transgenic plants (REM7-ind, ATHB25-ind, and ATHB25/REM7-ind) (in this supplemental text we represent the status of gene induction with "-ind" after the gene name, as done in the main manuscript), we produced more than 10 individual lines for each transgenic plant and performed gene expression analysis in the real-time quantitative reverse transcription PCR (RT-qPCR) [See the following section 'Real-time quantitative reverse transcription PCR (RT-qPCR)'] (Figure S2D).

To investigate the promoter activity of ATHB25 and REM7, promoter:GUS constructs were generated. We amplified DNA fragments upstream of the ATG sites of the ATHB25 and REM7 coding sequences (CDSs) for use as promoters and 5'-untranslated regions (5'-UTR) from Arabidopsis genomic DNA. The promoter sequences were amplified with the following primer sets (Table S2): ATHB25_promoter_FW and ATHB25_promoter_RV for ATHB25 promoter; REM7_promoter_FW and REM7_promoter_RV for REM7 promoter; attB1 and attB2 for the overhang sequences for the Gateway Cloning. The length of the promoter regions, including the 5'-UTR for both ATHB25 and REM7, was 3077 bp and 1250 bp, respectively. Each DNA fragment was subcloned into the pDONR 221 vector and then transferred into pGWB533 to generate promoter:GUS constructs, using the Gateway Cloning Kits (Invitrogen, USA)(Nakagawa et al., 2009).

We also produced 35S:RFP:CDS, pDEST-BTM116:CDS and pDEST-GAD424:CDS constructs to analyze the intracellular location and protein interactions of ATHB25 and REM7. The CDSs were also amplified and cloned, for overexpression of the RFP fusion protein, into pGWB555 (Nakagawa et al., 2009), and for protein interactions in a yeast two-hybrid assay into bait and prey vectors, pDEST-BTM116 and pDEST-GAD424 (Mitsuda et al., 2010), respectively.

To produce the ATHB25-ox and REM7-ox plants (in this supplemental text we represent the status of over expression of the gene with "-ox" after the gene name), we also cloned the coding regions of ATHB25 and REM7 into the pGWB502 vector using Gateway Cloning (Nakagawa et al., 2009). We produced more than 10 lines of

ATHB25-ox and REM7-ox plants and confirmed gene overexpression by RT-PCR. We crossed some of ATHB25-ox and REM7-ox lines (Figure 6).

Phenotypic measurement

Number of SSOs and lateral roots was counted under the microscope (Figure S5A and D). SSO width, root length, and leaf area were calculated from the photographic images by using an Image J software (https://imagej.nih.gov/ij/) (Figure S5B, C, and E).

Chlorophyll measurement

Roots (50–100 mg fresh weight) crushed in liquid nitrogen were homogenized in 500 μl of 80% (v/v) acetone. After recovering the supernatant by centrifugation at 12,000 x g for 5 min, a second extraction was performed on the precipitated debris with 500 μl of 80% (v/v) acetone. After mixing the supernatants from the first and the second extraction, which resulted in 1 ml of 80% (v/v) acetone extracts, the absorbance at 720, 663, 647 and 470 nm was measured with an Ultrospec 3100 pro Spectrophotometer (GE Healthcare Life Sciences, Pittsburgh, USA, https://www.gelifesciences.com). The chlorophyll concentration of the samples was calculated as below (Lichtenthaler, 1987).

$$Chl\ a = 12.25 * Absorbance_{663\ \mathrm{nm}} - 2.79 * Absorbance_{647\ \mathrm{nm}}$$
$$Chl\ b = 21.5 * Absorbance_{647\ \mathrm{nm}} - 5.10 * Absorbance_{663\ \mathrm{nm}}$$

Photosynthetic analysis

The third or fourth true leaves or roots from 21-day-old transgenic plants were dark-incubated on MS agar plates for 15 min. Effective quantum yields of photosystem II in plant tissues under varying intensities of actinic light were monitored with the IMAGING-PAM fluorometer (MAXI version) and IMAGING-WIN software (WALZ, Effeltrich, Germany, http://www.walz.com/). Measurement parameters for the fluorometer were as follows: measuring light intensity = 1, measuring light frequency = 2, damping = 1, gain = 1, saturation pulse intensity = 10, actinic light duration = 3 min.

Microscopic analysis

For observation of embryos, ovules were smashed in water and the embryos were collected with a small incubation basket (ø100 µm) (INTAVIS Bioanalytical Instruments AG, Cologne, Germany, www.intavis.com). The embryos were treated with a clearing solution of chloral hydrate, water, and glycerol (8:3:1, vol:vol). The developing embryos were observed under a differential interference contrast (DIC) microscope. For histology, sectioning was performed as described previously (Hirakawa et al., 2008). Samples for SSOs and corresponding regions were fixed in an FAA solution (ethanol: water: acetic acid: formalin = 50:40:5:5), dehydrated in a graded series of ethanol and embedded in Technovit 7100 resin (Heraeus Kulzer GmbH, Wehrheim, Germany, www.heraeus-kulzer.com) according to the manufacturer's instructions. Sections (1–2 µm) were cut with a microtome RM2165 (Leica Biosystems, Wetzler, Germany, www.leicabiosystems.com) and stained with 0.02% toluidine blue-O solution. For visualization of the xylem and phloem cells, the sections were stained with 0.02% safranin-O and 0.005% aniline blue solution (Thermo Fisher Scientific) and observed under a light microscope with a U-MWU2 mirror unit (Olympus, Tokyo, Japan, http://www.olympus-lifescience.com). For observation of *CUC* and *WUS* gene, the roots of the 1-week-old F1 seedlings of ATHB25/REM7-ind plants with *pCUC2*:*VENUS* and *pWUS*:*deRED* plants (Heisler et al., 2005; Reddy and Meyerowitz, 2005) generated by an artificial crossing were treated with/without 5 µM estradiol and were observed under a Zeiss LSM700 confocal microscope with a 5× objective or a 20× objective lens (ZEISS, Oberkochen, Germany, http://www.zeiss.com/). To detect the signals of VENUS and dsRED, 488 and 545 nm laser lines were used for excitation in conjunction with a 500-530 and 545-605 nm band-pass filters, respectively. For a rapid optical clearing for the fluorescence microscopy of *pCUC2*:*VENUS* and *pWUS*:*deRED* plants, ClearSee solutions were used according to the manufacturer's manual (FUJIFILM, Osaka Japan, https://www.fujifilm.com/) after the fixation with 4% formaldehyde solution.

Photography

The photographs of whole plants and SSOs were taken with a GR Digital III camera (RICOH, Tokyo, Japan, http://www.ricoh.com/). ZEISS Stemi 2000-C microscope with an Olympus DP20 (Olympus), ZEISS Axiovert 200M system, and Nikon ECLIPSE TS2 (Nikon, Tokyo, Japan, http://www.nikon.com) were used for stereoscopic

microscopy images. An Olympus IX83 microscope system (Olympus) was used for histochemical images. The fluorescent microscopy images of a cross-section of an SSO were taken using an Olympus IX83 microscope system, a Zeiss LSM700 system, and a Keyence Biozero BZ-8000 microscope (Keyence, Osaka, Japan, http://www.keyence.com) with a Nikon PlanFluor ELWD lens (Nikon) (Figure 3K).

Glucuronidase analysis

Embryos were isolated using the incubation basket described in the Microscopic analysis section in the main text. Intact plants, trimmed organs, and embryos were incubated with 80% cold acetone for 15 min and then rinsed with GUS buffer containing 50 mM phosphate buffer pH7.2, 0.5 mM $K_3[Fe(CN)_6]$ and 0.5 mM $K_4[Fe(CN)_6]$. The samples were incubated at 37ºC for 1 h with the GUS buffer containing 1 mM 5-bromo-4-chloro-3-indolyl-β-D-glucuronide cyclohexylammonium salt (X-Gluc) and then rinsed with 70% EtOH. A clearing solution of chloral hydrate described in the 'Microscopic analysis' section was used for the observation of the GUS staining.

RNA isolation

Seedlings germinated on agar plates without estradiol for one week were then grown on estradiol-containing plates for another week. The roots of the seedlings were harvested. Total RNA was isolated with an RNeasy Plant Mini Kit and treated with an RNase-Free DNase Set according to the manufacturer's manual (QIAGEN).

Real-time quantitative reverse transcription PCR (RT-qPCR)

Reverse transcription was performed on 1.0 µg of total RNA with a SuperScript III First-Strand Synthesis System (Thermo Fisher Scientific). Quantitative PCR was performed with the DyNAmo HS SYBR Green qPCR kit (Thermo Fisher Scientific) on the DNA Engine Opticon 2 System (MJ Research, Waltham, USA, http://mj-research.com). Measurements were normalized to the levels of ubiquitin 10 (UBQ10). The following gene-specific primer sets for *ATHB25, REM7*, and *UBQ10* were used (Table S3): ATHB25_RT-FW and ATHB25_RT-RV for *ATHB25*; REM7_RT-FW and REM7_RT-RV for *REM7*; UBQ10_FW and UBQ10_RV for *UBQ10*.

Microarray Analysis

Total RNA extracted from the roots was labeled with the Low Input Quick Amp Labeling Kit (one color, Cyanine3-CTP) (Agilent Technologies, Santa Clara, CA, USA, www.agilent.com) and hybridized to the Agilent Arabidopsis Oligo DNA Microarray Ver. 4 (Agilent Technologies) with the Gene Expression Hybridization Kit (Agilent Technologies), according to the manufacturer's manual. The labeling reaction was verified with the RNA Spike-In Kit (Agilent Technologies). The cDNA concentration and quality were confirmed with the NanoDrop ND-1000 spectrophotometer (Thermo Fisher Scientific) and Agilent 2100 Bioanalyzer (Agilent Technologies). The hybridized images were scanned with an Agilent Technology Scanner G2505C and were processed by Feature Extraction software v. 10.7.3.1 (Agilent). Normalization and analysis of microarray data were performed using GeneSpring GX software v. 12 (Tomy Digital Biology, Tokyo, Japan, www.digital-biology.co.jp) and R v. 3.2.1 (www.r-project.org) with Bioconductor, v. 3.1 (https://www.bioconductor.org). The data were normalized to the 75-percentile shift per chip to the median value of all samples. Microarray data for ATHB25-, REM7- and ATHB25/REM7-induced plants are available in the Gene Expression Omnibus (GEO) DataSets series with accession No. GSE105401 at the National Center for Biotechnology Information website (http://www.ncbi.nlm.nih.gov/gds). To draw heat-maps, the genes with more than 10-fold change values among treatments were chosen and z-scored (Data S1). Genefilter and gplots packages in R with Bioconductor generated the heat-maps. To investigate the expression of the ATHB25- and REM7-regulated genes during development, the array data from the gene expression map of *Arabidopsis thaliana* development (accession no. E-TABM-17) were downloaded from the public database, ArrayExpress (http://www.ebi.ac.uk/arrayexpress/experiments/E-TABM-17/) (Brazma et al., 2003; Schmid et al., 2005).

Data mining of microarray data

We explored and evaluated the genes downstream of *ATHB25* and *REM7* in DNA microarray experiments. A principal component analysis (PCA) which is a mathematical algorithm that visually assesses similarities and differences among

samples and determines whether the samples can be grouped, was performed in R (Ringner, 2008). A pseudo-count of 16 was added to the normalized values to calculate the log-fold changes (LogFC) and the z-scored LogFC was used for the PCA. GO enrichment analysis was performed with >10-fold regulated genes using the AmiGo2 browser (http://amigo.geneontology.org/amigo) on the Gene Ontology Consortium website (The Gene Ontology Consortium, 2015).

Protein interactions between ATHB25 and REM7.

Clonal transformed yeast cells (L40) harboring the ATHB25- or REM7- bait and prey plasmids were grown for three days at 30ºC in SC (synthetic-complete) liquid medium without U, W, and L amino acids. The cells were then transferred to SC medium plates without U, W, L and H, but with 1.5 mM 3-aminotriazole and were grown for a week at 22ºC. We did not observe any obvious yeast growth on the SC-UWLH plates, which strongly suggests there was no protein interaction between ATHB25 and REM7.

Supplemental Documents

Identification of the ATHB25 and REM7 genes that induce the SSO formation

We observed SSO formation in at least eight out of 11 lines that expressed the nine TF genes simultaneously under induction conditions. It is possible that some of these genes might be redundant. To evaluate these nine TFs as candidates for the establishment of SSOs, we produced several constructs that induced two or four out of the nine TF genes using the pGWB501_TOPX vector: set B (At3g61310 and At4g31805), set C (ATHB25 and REM7) and the combined set 'B and C' (At3g61310, At4g31805, ATHB25, and REM7) (Figure S2C). In the induction experiments using a series of constructs combining these TF genes, more than 80% of the 20 'set C' lines that simultaneously expressed both *ATHB25* and *REM7*, produced SSOs to the same extent as when all nine TF genes were co-induced (Figure 1: Figure S1). The combined set 'B and C' constructs also induced the SSO under the induction conditions, while the set B did not. Expression of either gene alone did not result in the formation of SSOs under normal induction conditions, although *ATHB25* formed SSOs after cytokinin application (Figure 2B). We did not analyze the set A construct. Therefore, we concluded that both *ATHB25* and *REM7* play an essential role in the formation of SSOs.

Annotation of the ATHB25 and REM7 families

The *ATHB25* gene encodes a zinc-finger homeodomain protein [HOMEOBOX PROTEIN 25 (ATHB25), ZINC FINGER HOMEODOMAIN 1 (ZHD1), ZINC FINGER HOMEODOMAIN 2 (ZFHD2)] (Figure S2E). A previous study reported that overexpression of *ATHB25* increases expression of *GIBBERELLIC ACID3-OXIDASE 2*, encoding a gibberellin (GA) biosynthetic enzyme, as well as the levels of GA_1 and GA_4 (Bueso et al., 2014). It was proposed that ATHB25 plays a role in gibberellin synthesis, related to seed longevity (Bueso et al., 2014). In Arabidopsis, there are 14 zinc-finger homeobox proteins belonging to the ZHD family that exhibit a putative zinc finger and a homeodomain (Tan and Irish, 2006) (the genes similar to ATHB25 are represented in Figure S2E). *ATHB22*, [*At4g24660*, *MATERNAL EFFECT EMBRYO ARREST 68* (*MEE68*), *ZINC FINGER HOMEODOMAIN2* (*ZHD2*)], a paralogue of *ATHB25*, controls embryo development, ending seed dormancy and cooperates with

ATHB25 in gibberellin synthesis. The double mutant involving *athb25* and *athb22* is reported to have decreased gibberellin. *ATHB21* [*At2g02540*, *ZINC FINGER HOMEODOMAIN 3* (*ZHD3*), *ZINC FINGER HOMEODOMAIN 4* (*ZFHD4*)] and *ATHB31* [*At1g14440*, *ZINC FINGER HOMEODOMAIN 4* (*ZHD4*), *FLORAL TRANSITION AT THE MERISTEM 2* (*FTM2*)] are also similar in structure to ATHB25 (Tan and Irish, 2006). *ATHB21* is expressed in vascular tissues, however its molecular function is unknown. *ATHB31* is proposed to regulate the shoot meristem during the transition from the vegetative to reproductive stage, controlling photoperiodic flowering. The other paralogues, *ATHB33* [*At1g75240*, *HOMEOBOX PROTEIN 33* (*ATHB33*), *ZINC-FINGER HOMEODOMAIN 5* (*ZHD5*)] and *ATHB29* [*At1g69600*, *ZINC FINGER HOMEODOMAIN 11* (*ZHD11*), *ZINC FINGER HOMEODOMAIN 1* (*ZFHD1*)] are known to mediate the abscisic acid-activated signaling pathway and the dehydration response, respectively (Tran et al., 2007). However, little is known about the function of the ZHD family. Arabidopsis has approximately 100 homeobox genes, many of which have been shown to play critical roles in various developmental processes. Other classes of homeobox proteins without zinc fingers, the Class I *knox* genes such as *SHOOT MERISTEMLESS* (*STM*) and *KNOTTED-LIKE HOMEOBOX*, the *WOX* class of HD-containing genes such as *STIMPY/WOX9* and *WUSCHEL* (*WUS*), and the Class III HD-Leu zipper proteins such as *PHAVOLUTA*, *PHABULOSA*, *REVOLUTA*, *ATHB8* and *ATHB15/CNA*, regulate the development and architecture of the embryo, vascular tissues, the SAM, leaves or flowers. Thus, sequence similarity between these genes suggests that ATHB25 regulates certain aspects of plant development, in addition to gibberellin synthesis and seed dormancy.

REM7 (*At3g18960*) is a gene belonging to the *REM* (*Reproductive Meristem*) gene family, which is a subgroup of the AP2/B3 transcription factors (Figure S2F) (Mantegazza et al., 2014). The *REM7* has been reported as one of the genes expressed in the reproductive meristem, however, it has not been functionally characterized to date. It is proposed that some of its paralogues may influence plant development and differentiation. For instance, the B3 domain proteins, VP1/ABI3-like 1 [VAL1, also termed as HIGH-LEVEL EXPRESSION OF SUGAR INDUCIBLE GENE 2 (HSI2), At2g30470] and VAL2/HSI1 (At4g32010), play important roles in stem cell

maintenance and cell differentiation. The sequence similarity between these genes suggests that *REM7* also plays a critical role in plant development.

Though some of these paralogues belonging to the ZHD and AP2/B3 families are known as the key factors in various aspects of plant development, it has not been reported about the function of both ATHB25 and REM7 on the shoot development. The molecular function of both genes and their paralogues largely remains unclear.

The downstream genes regulated in ATHB25/REM7-ind

We evaluated the genes downstream of *ATHB25* and *REM7* in DNA microarray experiments. The PCA showed that data from the plants with estradiol treatment were visualized as different groups in a distinct direction (Figure S9A), suggesting that the expression profile in the ATHB25/REM7-ind plants is not simply explained by the sum of that in each of the single ATHB25-ind and REM7-ind plants. This might be explained by the indirect effects of organ development. In comparison with the single ATHB25 induction, ATHB25/REM7-ind only developed the SSOs. Various indirect genes involved in the following events such as chloroplast development and gravitropism are misrelated in the ATHB25/REM7. Many genes upregulated in ATHB25-ind but not in ATHB25/REM7 may be involved only in the initial step of the SSO formation. Without the estradiol treatment, the components of ATHB25-ind, REM7-ind, and ATHB25/REM7-ind data were classified as the same cluster group.

Simultaneous induction of ATHB25 and REM7 promoted expression of SAM-specific genes [*WUS*, *STM*, *AGAMOUS-LIKE 15* (*AGL15*), *CUP-SHAPE COTYLEDON 1* and *3* (*CUC1* and *CUC3*), *REGULATOR OF AXILLARY MERISTEMS 1* and *2* (*RAX1* and *RAX2*) and *CLAVATA3* (*CLV3*)], the RM-quiescent center (QC) gene *WUSCHEL RELATED HOMEOBOX 5* (*WOX5*), the embryogenesis-specific gene *LEAFY COTYLEDON 1* (*LEC1*) and the wound-dependent callus-formation gene *WOUND INDUCED DEDIFFERENTIATION 1* (*WIND1*) (Figure S9B, Data S1). These upregulated genes are known to play important roles in the formation and maintenance of the SAM and in the induction of abnormal embryogenesis and callus. In contrast, simultaneous induction of ATHB25 and REM7 suppressed the repressors of root-

promoting genes, *TOPLESS* (*TPL*), the epigenetic repressor in polycomb repressive complex 1 (PRC1), *VALINE RESISTANT 1* (*VAL1*), *LATERAL SUPPRESSOR* (*LAS*), the RM-quiescent center (QC) specification gene *PLETHORA 1* (*PLT1*), the cytokinin-response gene *ARABIDOPSIS RESPONSE REGULATOR 1* (*ARR1*), the genes involved in lateral root formation *LOB DOMAIN PROTEIN 29* and *18* (*LBD29* and *LBD18*), the stress- and drought-related gene *DREB2A-INTERACTING PROTEIN2* (*DRIP2*), and the inhibitor of cyclin-dependent kinase *KIP-RELATED PROTEIN 3* (*KRP3*) (Figure S9B). These down-regulated genes are repressors of the cell cycle and of formation of lateral organs and roots. Curiously, *CUC* was induced, whereas *TPL* was repressed, in the roots of the ATHB25/REM7-ind plants. As the lack of *TPL* has been reported to promote root growth (Long et al., 2002), this expression of *CUC* and *TPL* seems inconsistent with the phenotypic data observed in the SSOs, in which stem-like organs are exhibited and, in the ATHB25/REM7-ox F1 plants, *cuc*-like phenotypes are displayed. As the ATHB25/REM7-ind plants were grown for a week after induction, the expression of *CUC*, *TPL*, and other downstream genes might contribute to these indirect effects as cell- or tissue-type markers, resulting in the observed tissue and organ characteristics of the SSOs.

Significantly enriched Gene Ontology (GO) analysis was performed to explore the biological processes of the targets of ATHB25 and REM7 (Table S4) (The Gene Ontology Consortium, 2015). “Regulation of vitamin metabolic process (GO: 0030656)”, “petal epidermis patterning (GO: 0080172)” and “regulation of L-ascorbic acid biosynthetic process (GO: 2000082)” were the categories that were most enriched in the up-regulated genes for the ATHB25/REM7-ind plants. These categories are associated with the regulation of chemical reactions and biosynthetic processes for vitamins or the coordinated growth and spatial arrangement of the cells. Regulation of response, transport, and biosynthesis of various secondary metabolites, such as ornithine, malate, cutin, t-RNA and carbon, which are generally synthesized during photosynthesis, were also associated with both TF induction. In the down-regulated genes from the ATHB25/REM7-ind plants, “response to fatty acid (GO: 0071398 and GO: 0070542)”, “ADP-ribosylation (GO: 0006471)”, “developmental programmed cell death (GO: 0010623)” and “DNA metabolism (GO: 0051103, GO: 0006273 and GO:

0006266)” were preferentially enriched. These categories appear to correspond to the ATHB25/REM7-ind plant phenotypes, which involve a high level of cell division and corpulence of the tissues. Therefore, it appears that simultaneous induction of both ATHB25 and REM7 controls a diverse range of biological processes mediating photosynthesis and cell growth.

Response to low oxygen, such as “response to hypoxia (GO: 0071456 and GO: 0001666)” or “response to oxygen levels (GO: 0071453, GO: 0036294, GO: 0070482 and GO: 0036293)” were processes predominantly enriched in the up-regulated genes from either the ATHB25-ind or REM7-ind plants. Some categories overlapped and were shared with those in the ATHB25/REM7-ind plants. It will be interesting to determine how the response to low oxygen levels mediates ATHB25 and REM7 function, triggering the SSO formation. Some of the categories enriched in the simultaneous induction of ATHB25 and REM7 plants (ATHB25/REM7-ind) were detected in neither ATHB25-ind nor REM7-ind plants. The GO analysis suggests that the phenotype displayed by the simultaneous induction of ATHB25 and REM7 is not caused by the sum of the individual functions of ATHB25 and REM7.

Spatiotemporal expressions of ATHB25 and REM7

To investigate the tissue- or organ-specific expression of the ATHB25 and REM7 genes, we produced plants containing an *ATHB25*- or *REM7*-promoter:*GUS* reporter gene construct. In GUS reporter assays, *ATHB25* expression was detected at the border between the shoot and the root in the embryo and at the basal region of the SAM of seedlings and mature plants (Figure S13A, C-F). *ATHB25* was also expressed in the vascular bundles at the borders between different tissues, such as the basal regions of the shoots and roots, leaf primordia and boundary domain of lateral roots (Figure S13G, H). In contrast, *REM7* expression was not observed in embryos (Figure S13J and K). *REM7* was detected in the veins of leaves around the SAM of seedlings and mature plants (Figure S13L, and M). The expression of *ATHB25* and *REM7* genes was partially but not always overlapped (Figure S13O). To investigate the intracellular localization, we produced the plants harboring the mRFP fusion constructs (35S Ω:mRFP:ATHB25 or REM7) described in the ‘plasmid construction’. Intracellular localization of both

ATHB25 and REM7 in wild-type plants resulted in their detection in the nucleus, consistent with the annotation of these genes as transcription factors (Figure S13I and N).

Physical interaction of ATHB25 and REM7

In a yeast two-hybrid system to assay direct protein-protein interactions(Mitsuda et al., 2010), no interactions between ATHB25 and REM7 proteins were observed (Figure S13P and Q). Due to the heterogenous system in yeast, it is still possible that these TFs interact together in plants. Comprehensive analyses of the physical interaction among the ATHB25 and REM7, and these paralogs will be required in a future study.

Loss-of-function phenotypes of ATHB25 and REM7

To investigate the loss-of-function phenotypes in plants disrupted both ATHB25 and REM7, we obtained the SALK T-DNA-tagged lines, SALK_133857C and SALK_014023C for ATHB25 and SALK_008105C for REM7, respectively, from the Salk Institute Genomic Analysis Laboratory (http://signal.salk.edu) (Alonso et al., 2003), and generated the double mutants (SALK_008105C x SALK_133857C, SALK_008105C x SALK_014023C) by classical crossing. The homologous F2 lines were confirmed by PCR. However, the double mutants generated by classical crossing with each T-DNA tagged line (Alonso et al., 2003) displayed no obvious alternations in the embryo maturation and SAMs of their phenotypes (Figure S13R-U). The redundancy of each paralogue might mask the mutation effects. The molecular functions of these genes and the paralogues remain unclear. Instead of *ATHB25* and *REM7* genes, these paralogues may play critical roles in shoot stem development.

SUPPLEMENTAL REFERENCES

Alonso, J.M., Stepanova, A.N., Leisse, T.J., Kim, C.J., Chen, H., Shinn, P., Stevenson, D.K., Zimmerman, J., Barajas, P., Cheuk, R., *et al.* (2003). Genome-wide insertional mutagenesis of Arabidopsis thaliana. Science *301*, 653-657.

Brazma, A., Parkinson, H., Sarkans, U., Shojatalab, M., Vilo, J., Abeygunawardena, N., Holloway, E., Kapushesky, M., Kemmeren, P., Lara, G.G., *et al.* (2003). ArrayExpress--a public repository for microarray gene expression data at the EBI. Nucleic Acids Res *31*, 68-71.

Bueso, E., Munoz-Bertomeu, J., Campos, F., Brunaud, V., Martinez, L., Sayas, E., Ballester, P., Yenush, L., and Serrano, R. (2014). ARABIDOPSIS THALIANA HOMEOBOX25 uncovers a role for Gibberellins in seed longevity. Plant Physiol *164*, 999-1010.

Chen, Q.J., Zhou, H.M., Chen, J., and Wang, X.C. (2006). A Gateway-based platform for multigene plant transformation. Plant Mol Biol *62*, 927-936.

Clough, S.J., and Bent, A.F. (1998). Floral dip: a simplified method for Agrobacterium-mediated transformation of Arabidopsis thaliana. Plant J *16*, 735-743.

Fujisawa, M., Takita, E., Harada, H., Sakurai, N., Suzuki, H., Ohyama, K., Shibata, D., and Misawa, N. (2009). Pathway engineering of Brassica napus seeds using multiple key enzyme genes involved in ketocarotenoid formation. J Exp Bot *60*, 1319-1332.

Heisler, M.G., Ohno, C., Das, P., Sieber, P., Reddy, G.V., Long, J.A., and Meyerowitz, E.M. (2005). Patterns of auxin transport and gene expression during primordium development revealed by live imaging of the Arabidopsis inflorescence meristem. Curr Biol *15*, 1899-1911.

Hirakawa, Y., Kondo, Y., and Fukuda, H. (2010). TDIF peptide signaling regulates vascular stem cell proliferation via the WOX4 homeobox gene in Arabidopsis. Plant Cell *22*, 2618-2629.

Hirakawa, Y., Shinohara, H., Kondo, Y., Inoue, A., Nakanomyo, I., Ogawa, M., Sawa, S., Ohashi-Ito, K., Matsubayashi, Y., and Fukuda, H. (2008). Non-cell-autonomous control of vascular stem cell fate by a CLE peptide/receptor system. Proc Natl Acad Sci U S A *105*, 15208-15213.

Huala, E., Dickerman, A.W., Garcia-Hernandez, M., Weems, D., Reiser, L., LaFond, F., Hanley, D., Kiphart, D., Zhuang, M., Huang, W., *et al.* (2001). The Arabidopsis

Information Resource (TAIR): a comprehensive database and web-based information retrieval, analysis, and visualization system for a model plant. Nucleic Acids Res *29*, 102-105.
Lichtenthaler, H.K. (1987). Chlorophylls and carotenoids: Pigments of photosynthetic biomembranes. Methods in Enzymology
148, 350-382.
Long, J.A., Woody, S., Poethig, S., Meyerowitz, E.M., and Barton, M.K. (2002). Transformation of shoots into roots in Arabidopsis embryos mutant at the TOPLESS locus. Development *129*, 2797-2806.
Mantegazza, O., Gregis, V., Mendes, M.A., Morandini, P., Alves-Ferreira, M., Patreze, C.M., Nardeli, S.M., Kater, M.M., and Colombo, L. (2014). Analysis of the arabidopsis REM gene family predicts functions during flower development. Ann Bot *114*, 1507-1515.
Mitsuda, N., Ikeda, M., Takada, S., Takiguchi, Y., Kondou, Y., Yoshizumi, T., Fujita, M., Shinozaki, K., Matsui, M., and Ohme-Takagi, M. (2010). Efficient yeast one-/two-hybrid screening using a library composed only of transcription factors in Arabidopsis thaliana. Plant Cell Physiol *51*, 2145-2151.
Moore, I., Samalova, M., and Kurup, S. (2006). Transactivated and chemically inducible gene expression in plants. Plant J *45*, 651-683.
Nakagawa, T., Ishiguro, S., and Kimura, T. (2009). Gateway vectors for plant transformation. Plant Biotechnology *26*, 275-284.
Obayashi, T., Okamura, Y., Ito, S., Tadaka, S., Aoki, Y., Shirota, M., and Kinoshita, K. (2014). ATTED-II in 2014: evaluation of gene coexpression in agriculturally important plants. Plant Cell Physiol *55*, e6.
Ogata, Y., Suzuki, H., Sakurai, N., and Shibata, D. (2010). CoP: a database for characterizing co-expressed gene modules with biological information in plants. Bioinformatics *26*, 1267-1268.
Reddy, G.V., and Meyerowitz, E.M. (2005). Stem-cell homeostasis and growth dynamics can be uncoupled in the Arabidopsis shoot apex. Science *310*, 663-667.
Rhee, S.Y., Beavis, W., Berardini, T.Z., Chen, G., Dixon, D., Doyle, A., Garcia-Hernandez, M., Huala, E., Lander, G., Montoya, M., *et al.* (2003). The Arabidopsis Information Resource (TAIR): a model organism database providing a centralized,

curated gateway to Arabidopsis biology, research materials and community. Nucleic Acids Res *31*, 224-228.

Ringner, M. (2008). What is principal component analysis? Nat Biotechnol *26*, 303-304.

Rosspopoff, O., Chelysheva, L., Saffar, J., Lecorgne, L., Gey, D., Caillieux, E., Colot, V., Roudier, F., Hilson, P., Berthome, R., *et al.* (2017). Direct conversion of root primordium into shoot meristem relies on timing of stem cell niche development. Development *144*, 1187-1200.

Schmid, M., Davison, T.S., Henz, S.R., Pape, U.J., Demar, M., Vingron, M., Scholkopf, B., Weigel, D., and Lohmann, J.U. (2005). A gene expression map of Arabidopsis thaliana development. Nat Genet *37*, 501-506.

Seki, M., Satou, M., Sakurai, T., Akiyama, K., Iida, K., Ishida, J., Nakajima, M., Enju, A., Narusaka, M., Fujita, M., *et al.* (2004). RIKEN Arabidopsis full-length (RAFL) cDNA and its applications for expression profiling under abiotic stress conditions. J Exp Bot *55*, 213-223.

Takita, E., Kohda, K., Tomatsu, H., Hanano, S., Moriya, K., Hosouchi, T., Sakurai, N., Suzuki, H., Shinmyo, A., and Shibata, D. (2013). Precise sequential DNA ligation on a solid substrate: solid-based rapid sequential ligation of multiple DNA molecules. DNA Res *20*, 583-592.

Tan, Q.K., and Irish, V.F. (2006). The Arabidopsis zinc finger-homeodomain genes encode proteins with unique biochemical properties that are coordinately expressed during floral development. Plant Physiol *140*, 1095-1108.

The Gene Ontology Consortium (2015). Gene Ontology Consortium: going forward. Nucleic Acids Res *43*, D1049-1056.

Tran, L.S., Nakashima, K., Sakuma, Y., Osakabe, Y., Qin, F., Simpson, S.D., Maruyama, K., Fujita, Y., Shinozaki, K., and Yamaguchi-Shinozaki, K. (2007). Co-expression of the stress-inducible zinc finger homeodomain ZFHD1 and NAC transcription factors enhances expression of the ERD1 gene in Arabidopsis. Plant J *49*, 46-63.

Zuo, J., Niu, Q.W., and Chua, N.H. (2000). Technical advance: An estrogen receptor-based transactivator XVE mediates highly inducible gene expression in transgenic plants. Plant J *24*, 265-273.
